# New Cladiellin-Type Diterpenoids from the South China Sea Soft Coral *Cladiella krempfi*: Structures and Molecular Docking Analysis in EGFRs

**DOI:** 10.3390/md20060381

**Published:** 2022-06-07

**Authors:** Yang Jin, Li-Gong Yao, Yue-Wei Guo, Xu-Wen Li

**Affiliations:** 1 School of Chinese Materia Medica, Nanjing University of Chinese Medicine, Nanjing 210023, China; j20-jinyang-nj@simm.ac.cn; 2 State Key Laboratory of Drug Research, Shanghai Institute of Materia Medica, Chinese Academy of Sciences, 555 Zu Chong Zhi Road, Zhangjiang Hi-Tech Park, Shanghai 201203, China; yaoligong@simm.ac.cn; 3 Drug Discovery Shandong Laboratory, Bohai Rim Advanced Research Institute for Drug Discovery, Yantai 264117, China

**Keywords:** soft coral, *Cladiella krempfi*, cladiellin-type diterpenoid, X-ray diffraction, structure-activity relationship

## Abstract

Two new cladiellin-type diterpenoids (**1** and **2**) and four known related compounds **3**–**6**, were isolated from the South China Sea soft coral *Cladiella krempfi*. Compound **2** is the third example of cladiellins of an unusual peroxy group in the C-6 position in *C. krempfi*. The structures and absolute configurations of the new compounds were established by extensive spectroscopic analysis, X-ray diffraction, and/or chemical correlation. In bioassay, all the compounds were evaluated for cytotoxicity and epidermal growth factor receptor (EGFR) inhibitory activity. A molecular docking experiment was conducted to study the structure–activity relationship of cladiellin-type diterpenoids on EGFR inhibitory activity.

## 1. Introduction

**Figure 1:**
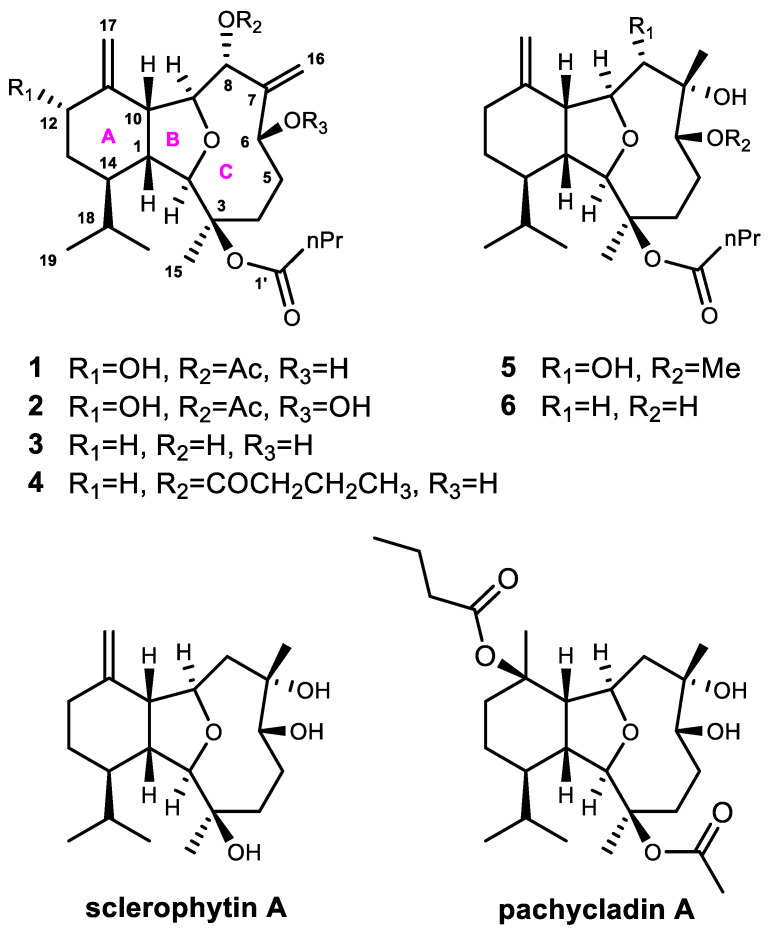
Structures of compounds **1**–**6** and two bioactive reference compounds sclerophytin A and pachycladin A.

Soft corals of the genus *Cladiella* (order Alcyonacea, family Alcyoniidae) are widely distributed in the tropical Indo-Pacific area [1]. They are rich sources of diverse and complex diterpenoids, especially 2,11-cyclized cembranoids, which are divided into three related structural classes: cladiellins (ether formation between C-2 and C-9, also known as eunicellins [2]), briarellins (ether formation between C-2/C-9 and C-3/C-20), and asbestinins (converted from the briarellin scaffold through a 11,12-methyl shift) [3]. A literature survey indicated that cladiellins isolated from *C. krempfi*, usually with signal ether bridge (C2–C9) [4,5], also have different oxidation patterns, such as two ether bridges (C-2/C-9 and C-3/C-7 or C-2/C-9 and C-12/C-19), or the esterification of C-19 [6,7,8]. Some of these diterpenoids also showed broad biological activities, such as anti-inflammatory [9], antifouling [10], antiproliferative [4], and cytotoxic effects [11]. Particularly, sclerophytin A showed significant cytotoxicity against mouse lymphocytic leukemia L1210 cells at a concentration as low as 1 ng/mL (Figure 1) [12]. Moreover, epidermal growth factor receptor (EGFR), related to the inhibition of tumor cell proliferation, angiogenesis, tumor invasion, metastasis, and apoptosis, is the only target protein found so far that cladiellin-type diterpenoids play a role in. For instance, Sayed and co-workers reported that pachycladin A exhibited promising EGFR inhibitory activity with an IC_50_ value of 0.5 μM (Figure 1) [13]. Therefore, the further investigation of cladiellins have attracted chemists and pharmacologists from all over the world [13,14].

In our ongoing efforts on the discovery of novel and bioactive marine natural products, several cladiellins have been isolated from soft corals [4,5,15]. In order to obtain more sclerophytin A and pachycladin A, such as new and bioactive cladiellins, *C. krempfi* was collected off Ximao Island, Hainan Province, China and chemically investigated, resulting in the isolation and characterization of two new cladiellin-type diterpenoids, namely litophynols C and D (**1** and **2**) (Figure 1) and four known ones (**3**–**6**). Herein, we report the isolation, structure elucidation, biological evaluation, and the preliminary structure–activity relationship (SAR) analysis of the isolates, assisted by the molecular docking experiment.

## 2. Results and Discussion

The Et_2_O-soluble portion of the acetone extract of the soft coral *C. krempfi* yielded the pure compounds **1** (1.4 mg), **2** (5.7 mg), **3** (2.2 mg), **4** (2.0 mg), **5** (2.1 mg), and **6** (1.0 mg), respectively. The known compounds **3**–**6** were readily identified as litophynol A (**3**) [16], 8-butyryl-litophynol A (**4**) [5], kremfielin B (**5**) [9], and litophynin E (**6**) [17] by comparing their NMR spectroscopic data and optical rotation with those reported in the literature.

Litophynol C (**1**) was isolated as an optically active colorless oil. Its molecular formula was established as C_26_H_40_O_7_ by HR-ESIMS (*m*/*z* 487.2671 [M + Na]^+^, calcd. 487.2666), indicating seven degrees of unsaturation. The ^13^C NMR, DEPT, and HSQC spectra of **1** revealed 26 carbon signals including five sp^3^ methyls, five sp^3^ methylenes, nine sp^3^ methines (five oxygenated ones at δ_C_ 91.1, 79.2, 83.7, 71.5, and 68.4), one oxygenated sp^3^ quaternary carbon (δ_C_ 84.7), two sp^2^ methylene, and four sp^2^ quaternary carbons (two ester carbonyls at δ_C_ 172.7 and 170.8). The diagnostic ^1^H and ^13^C NMR resonances, as well as coupling constants of the connected protons (Table 1), indicated the presence of two disubstituted terminal double bonds [δ_H_ 5.37, 5.64/δ_C_ 120.2 (CH_2_), δ_C_ 149.4 (qC); δ_H_ 4.89, 5.15/δ_C_ 116.4 (CH_2_), δ_C_ 146.1 (qC)]. Two double bonds and two ester carbonyls occupied four of the seven degrees of unsaturation, indicating that a tricyclic ring system belonged to the structure.

**Figure 2:**
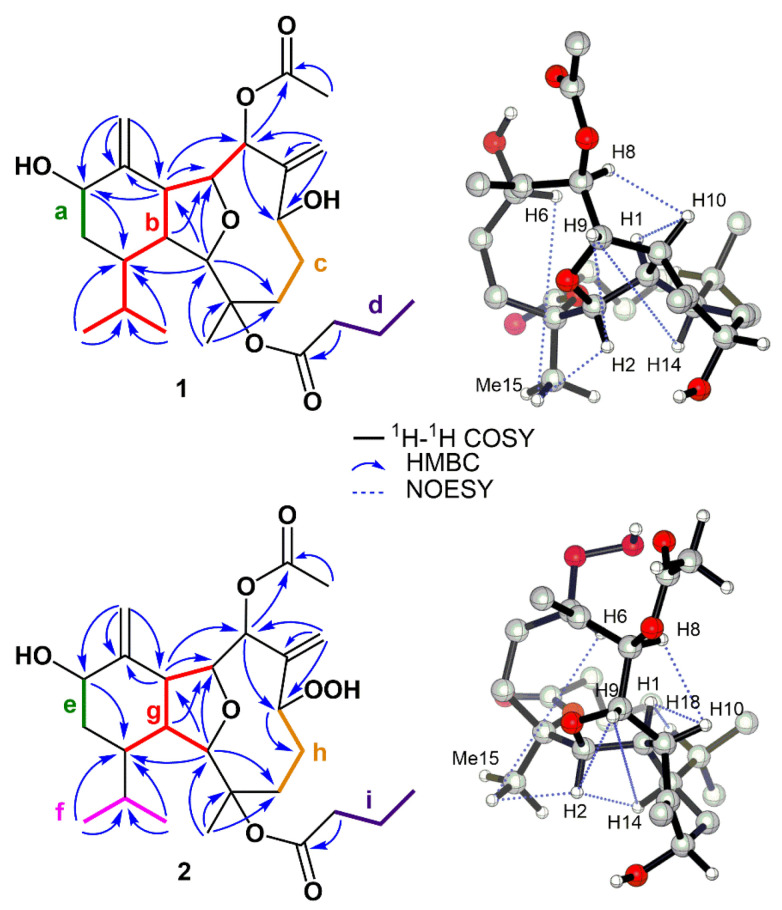
^1^H-^1^H COSY, key HMBC, and NOESY correlations of compounds **1** and **2**.

The planar structure of **1** was determined by ^1^H-^1^H COSY and HMBC experiments (Figure 2). Four structural fragments **a**–**d** were rapidly identified by carefully analyzing the ^1^H-^1^H COSY spectrum of **1**, with the clear correlations of H-12 (δ_H_ 4.46)/H_2_-13 (δ_H_ 1.27, 1.94) (**a**); H-8 (δ_H_ 5.28)/H-9 (δ_H_ 4.68)/H-10 (δ_H_ 2.94)/H-1 (δ_H_ 2.23)/H-14 (δ_H_ 1.87)/H-18 (δ_H_ 1.89)/H_3_-19 (δ_H_ 0.99)/H_3_-20 (δ_H_ 0.76) (**b**); H_2_-4 (δ_H_ 1.79, 2.23)/H_2_-5 (δ_H_ 1.74, 2.20)/H-6 (δ_H_ 4.71) (**c**); H_2_-2′ (δ_H_ 2.14)/H_2_-3′ (δ_H_ 1.58)/H_3_-4′ (δ_H_ 0.92) (**d**), respectively. The connection between fragments **a**–**d** was further figured out by the detailed interpretation of the well-resolved HMBC spectrum (Figure 2).

The HMBC correlations from H-10 to C-11/C-12, from H_2_-17 to C-10/C-11/C-12, and from H-12 to C-14 revealed the presence of a cyclohexane ring (ring A) with a terminal double bond at C-11 and an isopropyl group at C-14. The cross peaks from H_3_-15 to C-2/C-3/C-4 and from H-2 to C-4/C-9/C-10/C-14 revealed that ring A and fragment **c** were connected via C-2 and C-3. Additionally, the cross peaks from H_2_-16 to C-6/C-7/C-8 suggested a cyclodecane ring fused with ring A at C-1 and C-10. Furthermore, the strong correlation from H-2 to C-9 and the remaining degree of unsaturation all indicated an ether bridge between C-2 and C-9, which divided the cyclodecane ring into a tetrahydrofuran ring B and an oxocane ring C. Finally, the acetyl group at C-8 was deduced by the HMBC correlation from H-8 to C-1′′ and from H_3_-2′′ to C-1′′, which also suggested that the connection of the rest butyryl group should be at C-3. Thus, the planar structure of **1** was determined as shown in Figure 1.

The relative configuration of **1** was established by analysis of its NOESY spectrum (Figure 2). As the orientation of H-1 in all the reported cladiellin-type diterpenoids was determined as *β* [17], that of compound **1** was also arbitrarily assigned as s *β*-configuration. The NOE correlation of H-1/H-10 and H-10/H-8 suggested that H-8 and H-10 were all *β*-configurations. In addition, the cross peaks of H-6/H_3_-15, H_3_-15/H-2, H-2/H-9, and H-9/H-14 indicated the *α*-configuration in H-6, Me-15, H-2, H-9, and H-14. Above all, the relative configuration of **1** was ambiguously deduced as 1R*, 2R*, 3R*, 6R*, 8R*, 9S*, 10R*, and 14R*. Due to the confusing NOE correlations between adjacent H signals, the orientation of the rest of H-12 was still hard to define.

**Figure 3:**
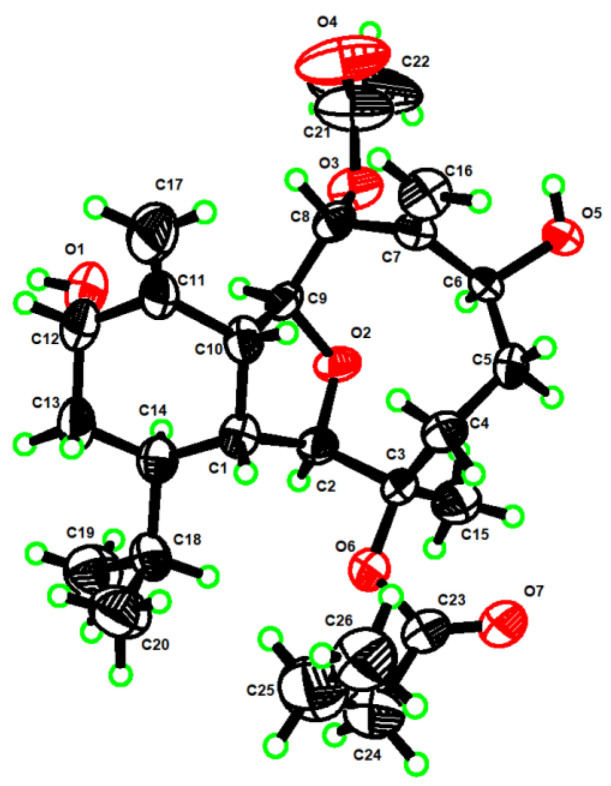
Perspective ORTEP drawing of X-ray structures of **1** (displacement ellipsoids are drawn at the 50% probability level).

In order to confirm the absolute configuration of **1**, we tried with much effort and fortunately obtained its single crystal from the recrystallization of **1** in methanol, which allowed a successful performance of X-ray crystallography study using Cu Kα radiation (λ = 1.54178 Å). Analysis of the X-ray data unambiguously confirmed the planar structure of **1**, and determined its absolute configuration as 1*R*, 2*R*, 3*R*, 6*R*, 8*R*, 9*S*, 10*R*, 12*S*, 14*R* [Flack parameter was 0.04(13)] (Figure 3). Thus, the structure of **1** was determined, namely litophynol C (Figure 1).

Litophynol D (**2**) was isolated as an optically active colorless oil. Its molecular formula was established as C_26_H_40_O_8_ by HR-ESIMS (*m*/*z* 503.2618 [M + Na]^+^, calcd. 503.2615), indicating the presence of seven degrees of unsaturation. The ^13^C NMR, DEPT, and HSQC spectra of **2** revealed 26 carbon signals, including five sp^3^ methyls, five sp^3^ methylenes, nine sp^3^ methines, one oxygenated sp^3^ quaternary carbon, two sp^2^ methylene, and four sp^2^ quaternary carbons. In fact, as shown in Table 1, the NMR data of **2** were extremely close to those of **1**, indicating that they were structure analogs. After the careful comparison of 1D and 2D NMR spectra, the main differences between **1** and **2** were the deshielding of C-6 from δ_C/H_ 68.4/4.71 in **1** to 81.6/4.93 in **2**. The chemical shifts in the C-6 surrounding carbons also changed slightly, e.g., C-5 from δ_C/H_ 35.9/1.74, 2.20 in **1** to 30.4/1.50, 2.16 in **2**; C-7 from δ_C_ 149.4 in **1** to 145.0 in **2**, which strongly implied the hydroxyl at C-6 in **1** was replaced by the hydroperoxide group in **2**. The 16 mass units more of molecular weight in **2** further supported our determination. The detailed 2D NMR analyses, as shown in Figure 2, confirmed the planar structure and relative configuration of **2.**

**Scheme 1:**
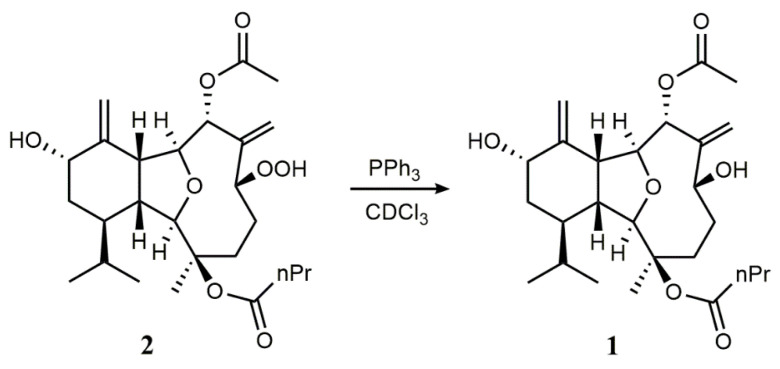
Reduction of **2** to **1**.

**Figure 4:**
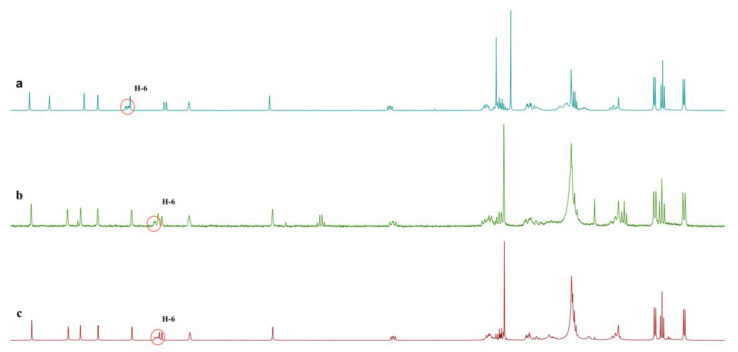
Comparison of ^1^H NMR spectra (recorded at 600 MHz in CDCl_3_): (**a**) ^1^H NMR spectrum of **2**; (**b**) ^1^H NMR spectrum of mixture after reaction; (**c**) ^1^H NMR spectrum of **1**.

To further confirm our assignment, triphenylphosphine was used to convert the hydroperoxide group of **2** into the corresponding alcohol in CDCl_3_ (Scheme 1). The reduction of **2** resulted in a compound identical [^1^H NMR (Figure 4) and MS data (Figure S30)] to **1**. Thus, the absolute configuration of **2** was unambiguously determined to be the same as that of **1** (Figure 1).

Considering the interesting anti-cancer activities displayed by sclerophytin A and pachycladin A [12,13], all isolates were used to carry out the tests of cytotoxic effects on A549 (human lung cancer) tumor cells and EGFR inhibitory activities; however, none of them showed obvious bioactivities (IC_50_ > 20 μM). By comparing the structures of our isolated compounds with those of the most bioactive, sclerophytin A and pachycladin A, it seemed that the esterification of the hydroxy groups, the site of the esterification, and the length of the ester influenced the activity. Therefore, the selective hydrolysis of the isolated compounds might result in some sclerophytin A and pachycladin A, which could be anti-cancer drug candidates. However, the scarcity of the isolates prevented further chemical correlation. We then tried to do an intensive molecular docking analysis to study the SAR of these types of molecules on EGFRs, aiming to give an insight into future structural modification.

**Figure 5:**
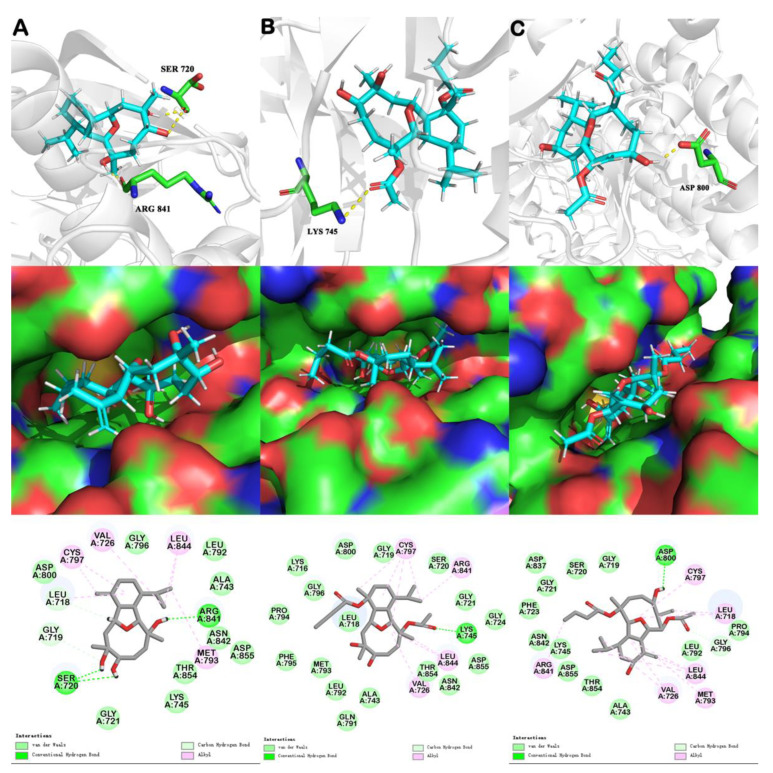
In silico binding mode of sclerophytin A, pachycladin A, and litophynol C (**1**) at EGFR kinase crystal structure 5X2A: upper row—the transparent protein surface, in light grey color, and three compounds shown as sticks with atoms colored C cyan, N blue, O red, and H white, are shown to emphasize the clear combination of hydrogen bonds within the target pocket; middle row—surfaces of 5X2A with combined compounds; lower row—two-dimensional ligand interaction diagrams of three compounds at the EGFR kinase domain. Left list (**A**) represents docking results of sclerohpytin A; Middle list (**B**) represents docking results of pachycladin A; Right list (**C**) represents docking results of litophynol C (**1**).

On the basis of the speculation on the SAR, molecular docking analysis was performed using all the isolated compounds, together with sclerophytin A and pachycladin A, on EGFRs. Since the structures of litophynol C (**1**) and pachycladin A were more similar than other isolated compounds, litophynol C (**1**) was selected for the detailed comparison with sclerophytin A and pachycladin A (Figure 5, the docking results of compounds **2**–**6** are added in Figure S31 and the binding energy of all the docking molecules are listed in Table S2 in Supplemetary Materials). The highly resolved EGFR crystal structure (PDB codes: 5X2A with a resolution of 1.85Å) was used to investigate the possible binding modes of the three compounds within the catalytic domain of the EGFR by means of the Discovery Studio software (Figure 5). Pachycladin A occupied the same region as the ligand of 7XO, which is a member of the SKLB compound series targeting EGFR activation [18], and its C-3 acetate participated in hydrogen bonds with Lys745, Asn842, and Asp855 of the EGFR crystal structures 5X2A (Figure 5B, upper row). Interestingly, Lys745 lay in the active site of the kinase. Sclerophytin A, Ser720, and ARG841 were combined by three hydrogen bonds, while the C-6 hydroxy of litophynol C (**1**) participated in hydrogen bond with Asp800, which did not lie in the active site (Figure 5A,C, upper rows). Furthermore, the C-3 acetate and C-11 butyrate of pachycladin A fully occupied the hydrophobic pocket, which promoted Van der Waals interactions with Gly719, Ser720, Gly721, Val726, Ala743, Gly796, Leu799, Asp800, and Thr854 (Figure 5B, mid and lower row). The lower binding affinity of compound **1**, compared to that of pachycladin A (Table 2), could be deemed to be related to the position and the length of the ester, which might influence the interaction with the hydrophobic pocket and explained why our isolates did not show obvious EGFR inhibitory activity. Although sclerophytin A has been previously reported to be super cytotoxic against mouse lymphocytic leukemia L1210 cells, it displayed less binding affinity than pachycladin A, with lower binding energy (Table 2), indicating that it might be less effective on non-small cell lung cancer; however, further validation and target fishing of this would be worthwhile.

## 3. Materials and Methods

### 3.1. General Experimental Procedures

1D and 2D NMR spectra were recorded on a Bruker AVANCE III 500 (125 MHz, Bruker Biospin AG, Fallanden, Germany) and Agilent 1260 Prospekt 2 Bruker Ascend 600 (600 MHz, Bruker Biospin AG, Fallanden, Germany) in CDCl_3_ using solvent signals as internal standards (CHCl3, δ_H_ 7.26 ppm; δ_C_ 77.16 ppm). IR spectra were recorded on a Nicolet 6700 spectrometer (Thermo Scientific, Waltham, MA, USA) with a KBr ATR plate. CD spectra were recorded on a J-815 instrument. Optical rotations were measured on a Perkin-Elmer 241-MC polarimeter (PerkinElmer, Fremont, CA, USA). Melting points were measured on an X-4 digital micromelting point apparatus. LR-ESIMS and HR-ESIMS data were recorded on a Bruker Daltonics Esquire 3000 plus instrument (Bruker Daltonics K. K., Kanagawa, Japan) and a Waters Q-TOF Ultima mass spectrometer (Waters, Milford, MA, USA). Commercial silica gel (100–200, 200–300 and 300–400 mesh; Qingdao, China) and Sephadex LH-20 gel (Amersham Biosciences, London, UK) were used for column chromatography (CC). Precoated SiO_2_ plates (HSGF-254, Yan Tai Zi Fu Chemical Group Co. Yantai, China) were used for analytical TLC. Semi-preparative HPLC was performed on an Agilent-1260 system equipped with a DAD G1315D detector at 210 and 254 nm using ODS-HG-5 (250 mm × 9.4 mm, 5 μm) by eluting with a CH_3_CN-H_2_O system at 3 mL/min. All solvents used for CC and HPLC were of analytical grades (Shanghai Chemical Reagents Co., Ltd., Shanghai, China) and chromatographic grade (Dikma Technologies Inc., Shanghai, China), respectively.

### 3.2. Biological Material

The soft coral specimen, identified as *C. krempfin* by Prof. Xiu-Bao Li from Hainan University, was collected along the coast of Ximao Island (east longitude 109°22′5.9″ and northern latitude 17°110′49.7″), Hainan Province, China in 2019, at a depth of 15 m. A voucher specimen (NO. 19XD-6) is available for inspection at the Shanghai Institute of Materia Media, Chinese Academy of Sciences.

### 3.3. Extraction and Isolation

The freeze-dried animals (96 g, dry weight) were cut into pieces and exhaustively extracted with acetone at room temperature (5 × 1 L). The acetone extract was evaporated to yield a brown residue, which was then partitioned between Et_2_O and H_2_O. The upper layer was concentrated under reduced pressure to obtain a brown residue 3.6 g, which was separated by gradient silica gel column chromatography (200–300 mesh, 0% to 100% Et_2_O in petroleum ether), yielding ten fractions (A–J). Fraction D (182.8 mg) was eluted by Sephadex LH-20 with PE/DCM/MeOH (2:1:1) to give five subfractions (D1–D5). Compound **5** (2.1 mg, t_R_ = 9.1 min) was obtained from the D2 fraction by RP-HPLC (CH_3_CN-H_2_O, 80:20, 3.0 mL/min). Fraction E (63.0 mg) was separated by Sephadex LH-20 (PE/DCM/MeOH, 2:1:1) to obtain four subfractions (E1–E4). Each of the subfractions E2–E4 were further purified by RP-HPLC under the same elution gradient (CH_3_CN-H_2_O, 80:20, 3.0 mL/min). The former subfraction E2 yielded compound **4** (2.0 mg, t_R_ = 21.5 min). Compound **1** (1.4mg, t_R_ = 6.8 min) was obtained from the midst subfraction E3. The latter subfraction E4 gave compound **2** (5.7mg, t_R_ = 7.4 min). Fraction F (220.2 mg) was analyzed by a column of Sephadex LH-20 eluted with CH_2_Cl_2_ to obtain six subfractions (F1–F6). Compound **6** (1.0 mg, t_R_ = 9.4 min) was obtained from subfraction F3. Compound **3** (2.2 mg, t_R_ = 9.0 min) was obtained by series of CC from fraction G (363.6 mg).

Litophynol C (**1**). Colorless crystal, mp 183–184 °C; ${\left[\alpha \right]}_{D}^{20}$ 24.9 (c 0.14, CHCl_3_); IR (KBr) v_max_/cm^−1^: 3471, 2924, 2353, 1725, 1254, 1071, 1031; for ^1^H NMR (CDCl_3_, 400 MHz) and ^13^C NMR (CDCl_3_, 125MHz) spectral data, see Table 1; HR-ESIMS m/z 487.2671 [M + Na]^+^ (calcd. for C_26_H_40_O_7_, 487.2666).

Litophynol D (**2**). Colorless oil; ${\left[\alpha \right]}_{D}^{20}$ 15.8 (c 0.57, CHCl_3_); IR (KBr) v_max_/cm^−1^: 3455, 2950, 1724, 1249, 1069, 1034; for ^1^H NMR (CDCl_3_, 400 MHz) and ^13^C NMR (CDCl_3_, 125MHz) spectral data, see Table 1; HR-ESIMS m/z 503.2618 [M + Na]^+^ (calcd. for C_26_H_40_O_8_, 503.2615).

## 4. *X*-ray Crystal Structure Analysis of 1

C_26_H_40_O_7_, M_r_ = 464.58, monoclinic, crystal size 0.15 × 0.08 × 0.05 mm^3^, space group P2_1_, a = 6.3306(2) Å, b = 43.1346(13) Å, c = 10.1469(3) Å, V = 2643.23(14) Å^3^, Z = 4, ρ_calcd_ = 1.167 g/cm^3^, F(000) = 1008.0, 22,999 collected reflections, 10,102 independent reflections (R_int_ = 0.0671, R_sigma_ = 0.0823), final R_1_ = 0.1189 (wR_2_ = 0.3028) reflections with I ≥ 2σ (I), R_1_ = 0.1270, wR_2_ = 0.3080 for all unique data. The X-ray measurements were made on a Bruker D8 Venture X-ray diffractometer with Cu Kα radiation (λ = 1.54178 Å) at 170.0 K. The structure was solved with the ShelXT [19] structure solution program using intrinsic phasing and refined with the ShelXL [20] refinement package using least squares minimization. Crystallographic data for 1 were deposited at the Cambridge Crystallographic Data Centre (Deposition nos. CCDC 2179835). Copies of these data can be obtained free of charge via www.cdcc.cam.ac.uk/conts/retrieving.html or from the Cambridge Crystallographic Data Centre, 12 Union Road, Cambridge CB21EZ, UK. [Fax: (+44) 1223-336-033. E-mail: deposit@ccdc.cam.ac.uk.]

## 5. Bioassay Procedures

### 5.1. EGFR Activity Assays

Six compounds were screened on EGFRs using staurosporine obtained from MedChemExpress (Cat. No. HY-15141; Lot. No. 41248) as the reference compound. In the bioassay for the EGFRs, testing kinases were performed in 1× kinase base buffer containing 50 mM HEPES (pH 7.5) and 0.0015% Brij-35, and stop buffer containing 100 mM HEPES (pH 7.5), 0.0015% Brij-35, 0.2% Coating Reagent #3, and 50 mM EDTA. For the compound preparing part, compounds were diluted to 50X of the final desired highest inhibitor concentration in the reaction by 100% DMSO, and 100 µL of the compound dilution was transferred to a well in a 96-well plate. Additionally, 100 µL of 100% DMSO was added to two empty wells for a no-compound control and a no-enzyme control in the same 96-well plate, which was marked as the source plate. Then, 10 µL of compound from the source plate was transferred to a new 96-well plate as the intermediate plate where each well contained 90 µL of 1× kinase buffer. The compounds were mixed in an intermediate plate for 10 min on a shaker. For the assay plate, 5 µL of each well from the 96-well intermediate plate was transferred to a 384-well plate in duplicates. For the kinase reaction, 2.5× enzyme solution and 2.5× peptide solution were prepared. Moreover, 5 µL of compound were settled in the assay plate with 10% DMSO. Additionally, 10 µL of 2.5× enzyme solution was added to each well of the 384-well assay plate that was incubated at room temperature for 10 min. Then, 10 µL of 2.5× peptide solution was added to each well of the 384-well that was incubated at 28 °C for 30 min and stopped by 25 µL stop buffer. The percentage of inhibition was estimated using max-conversion divided by the max-min, and inhibition values were converted by convert conversion values from the caliper program. IC50 values were obtained from fitting the data in the XLfit excel add-in version 4.3.1.

### 5.2. Anti-Tumor Assays

Anti-tumor assays were carried out using A549 (Human lung cancer) tumor cells (ATCC, #CCL-185), following a previously described procedure for a modification of the MTT colorimetric method [21,22], with 5-fluorouracil used as the positive control. To measure the anti-proliferative activity of tested compounds, three concentrations with three replications were performed on the cell line.

## 6. Molecular Docking

The cocrystal structure of an EGFR (PDB code 5X2A) was obtained from the RCSB Protein Data Bank. The water molecules were removed in Discovery Studio (DS), while the binding site of sclerophytin A, pachycladin A, and litophynol C (**1**) on the EGFR was consistent with that of 7XO. Moreover, the combined spherical area was x = 3.789, y = 17.478, z = −29.936, radius = 10.85. A clean protein tool was used to remove protein conformation, supplement incomplete amino acid disability, and refine the hydrogen, and the receptor was prepared for docking. Ligands were sketched in the ChemBioDraw program and uploaded to DS. Furthermore, optimized ligands were obtained through prepared ligand tools and minimized ligand tools. The molecular docking module (CDOCKER) was used for docking, and the simulation with the optimal situation of -CDOCKER ENERGY and -CDOCKER INTERACTION ENERGY scores was analyzed and visualized in Pymol [23,24]. Before calculating the binding energies, all ligands were minimized by an in situ ligand minimization tool. Under the calculated binding-energies tool, Poisson–Boltzamann with non-polar surface area (PBSA) was used to calculate the binding energy (salt concentration = 0.15, rest parameters were default).

## 7. Conclusions

In summary, this was the first detailed chemical investigation of *C. krempfi* from Ximao island in South China Sea. Two new cladiellin-type diterpenoids, litophynols C and D (**1** and **2**), and four known related compounds **3**–**6** were isolated and fully elucidated. The stereochemistry of the new compounds was unambiguously determined by extensive spectroscopic analysis, X-ray diffraction analysis, and/or chemical correlation. The discovery of **1** and **2** expanded the diversity and complexity of marine diterpenes. In bioassay, the results obtained were somewhat disappointing since none of the tested compounds are bioactive, while a preliminary SAR of these type of molecules, both isolated and previously reported ones, were studied with the assistance of molecular docking analysis. The above evidence indicated that the most bioactive compound, sclerophytin A, should be regarded as the starting material for further structural modification. It is worth mentioning that this molecule was totally synthesized by Yang and co-workers [10], which enabled further structural modification, and our biological study and SAR analysis, together with those previously reported by Sayed and co-workers [13,25], gave some information to avoid the synthesis of inactive compounds, such as compounds **1**–**6** related esters.

## Figures and Tables

**Table 1:** ^1^H NMR (δ_H_) and ^13^C NMR (δ_C_) data for **1** and **2** in CDCl_3_.

No.	1		2	
	δ_H_ (mult., *J*, Hz) ^a^	δ_C_ (mult) ^b^	δ_H_ (mult., *J*, Hz) ^a^	δ_C_ (mult) ^b^
1	2.23, m	43.8, d	2.24, m	43.8, d
2	3.84, s	91.1, d	3.86, s	91.0, d
3		84.7, s		84.5, s
4a	1.79, m	29.8, t	1.88, m	29.4, t
4b	2.23, m		2.26, m	
5a	1.74, m	35.9, t	1.50, m	30.4, t
5b	2.20, m		2.16, m	
6	4.71, d (8.1)	68.4, d	4.93, dd (3.6, 11.3)	81.6, d
7		149.4, s		145.0, s
8	5.28, s	79.2, d	5.25, s	78.6, d
9	4.68, d (10.5)	83.7, d	4.65, d (10.7)	83.8, d
10	2.94, dd (7.7, 10.9)	46.6, d	2.96, dd (7.7, 11.1)	46.4, d
11		146.1, s		145.8, s
12	4.46, s	71.5, d	4.47, s	71.5, d
13a	1.27, m	30.9, t	1.29, m	31.1, t
13b	1.94, m		1.92, m	
14	1.87, m	35.6, d	1.89, m	35.5, d
15	1.60, s	22.7, q	1.61, s	22.7, q
16a	5.37, s	120.2, t	5.51, s	121.6, t
16b	5.64, s		5.66, s	
17a	4.89, brs	116.4, t	4.91, brs	116.7, t
17b	5.15, brs		5.15, brs	
18	1.92, m	27.2, d	1.89, m	27.2, d
19	0.98, d (7.0)	21.8, q	0.99, d (6.9)	21.8, q
20	0.76, d (6.8)	15.5, q	0.76, d (6.8)	15.4, q
1′		172.7, s		172.7, s
2′	2.13, m	37.5, t	2.14, m	37.5, t
3′	1.59, m	18.6, t	1.58, m	18.6, t
4′	0.93, t (7.4)	13.8, q	0.92, t (7.4)	13.8, q
1′′		170.8, s		171.3, s
2′′	2.11, s	21.6, q	2.06, s	21.6, q

^a^ Recorded at 600 MHz. ^b^ Recorded at 125 MHz. Assignments were deduced by analysis of 1D and 2D NMR spectra.

**Table 2:** In silico docking parameters between sclerophytin A, pachycladin A, litophynol C, and the ligand in the 7XO site of 5X2A.

EGFR Crystal Structure	Compound ID	Number of Hydrogen Bonds	Binding Energy (kcal mol^−1^)	Van der Waals Energy (kcal mol^−1^)	-CDOCKER ENERGY (kcal mol^−1^)	-CDOCKER INTERACTION ENERGY (kcal mol^−1^)
5X2A	sclerophytin A	3	–1.74	–10.52	–31.78	43.13
	pachycladin A	1	–12.68	–15.40	–10.59	49.68
	litophynol C (**1**)	1	8.57	–17.40	–26.10	51.95

## Data Availability

Data are contained within the article or Supplementary Material.

## Supplementary Materials

The following supporting information can be downloaded at: https://www.mdpi.com/article/10.3390/md20060381/s1, Table S1: EGFR inhibitory data of compounds **1**–**6** and positive control, Table S2: In silico docking parameters between compounds **2**–**6** and the ligand of 7XO site of 5X2A, Figure S1: ^1^H NMR spectrum of compound **1** (600 MHz, CDCl_3_), Figure S2: ^13^C NMR spectrum of compound **1** (125 MHz, CDCl_3_), Figure S3: DEPT135 spectrum of compound **1** (125 MHz, CDCl_3_), Figure S4: HSQC spectrum of compound **1** (600 MHz, CDCl_3_), Figure S5: ^1^H-^1^H COSY spectrum of compound **1** (600 MHz, CDCl_3_), Figure S6: HMBC spectrum of compound **1** (600 MHz, CDCl_3_), Figure S7: NOESY spectrum of compound **1** (600 MHz, CDCl_3_), Figure S8: HR-ESI-MS spectrum of compound **1**, Figure S9: IR spectrum of compound **1**, Figure S10: ORD spectrum of compound **1**, Figure S11: ^1^H NMR spectrum of compound **2** (600 MHz, CDCl_3_), Figure S12: ^13^C NMR spectrum of compound **2** (125 MHz, CDCl_3_), Figure S13: DEPT135 spectrum of compound **2** (125 MHz, CDCl_3_), Figure S14: HSQC spectrum of compound **2** (600 MHz, CDCl_3_), Figure S15: ^1^H-1H COSY spectrum of compound **2** (600 MHz, CDCl_3_), Figure S16: HMBC spectrum of compound **2** (600 MHz, CDCl_3_), Figure S17: NOESY spectrum of compound **2** (600 MHz, CDCl_3_), Figure S18: HR-ESI-MS spectrum of compound **2**, Figure S19: IR spectrum of compound **2**, Figure S20: ORD spectrum of compound **2**, Figure S21: ^1^H NMR spectrum of compound **3** (400 MHz, CDCl_3_), Figure S22: ^13^C NMR spectrum of compound **3** (125 MHz, CDCl_3_), Figure S23: ^1^H NMR spectrum of compound **4** (400 MHz, CDCl_3_), Figure S24: ^13^C NMR spectrum of compound **4** (125 MHz, CDCl_3_), Figure S25: ^1^H NMR spectrum of compound **5** (500 MHz, CDCl_3_), Figure S26: ^13^C NMR spectrum of compound **5** (125 MHz, CDCl_3_), Figure S27: ^1^H NMR spectrum of compound **6** (500 MHz, CDCl_3_), Figure S28: ^13^C NMR spectrum of compound **6** (125 MHz, CDCl_3_), Figure S29: ^1^H NMR spectrum of mixture (600 MHz, CDCl_3_), Figure S30: HR-ESI-MR spectrum of mixture, Figure S31: In silico binding mode of compounds **2**–**6** at EGFR kinase crystal structure 5X2A.
