# Supplementary material for: New Cladiellin-Type Diterpenoids from the South China Sea Soft Coral Cladiella krempfi: Structures and Molecular Docking Analysis in EGFRs
Source: Mar Drugs. 2022 Jun 7;20(6):381. doi: 10.3390/md20060381 (PMC9229255; doi:10.3390/md20060381)
Supplement: Supplementary file 1 [file marinedrugs-20-00381-s001.zip › marinedrugs-1727765-supplementary.pdf]

New Cladiellin-type Diterpenoids from the South China Sea Soft Coral *Cladiella krempfi*: Structures and Molecular Docking analysis in EGFRs

Yang Jin,[1,3] Li-Gong Yao,[2,3] Yue-Wei Guo [1,2,3,*] Xu-Wen Li [1,2,3,*]

[1] *School of Chinese Materia Medica, Nanjing University of Chinese Medicine, 210023 Nanjing, China*

[2] *Drug Discovery Shandong Laboratory, Bohai Rim Advanced Research Institute for Drug Discovery, Yantai, 264117, China*

[3] *State Key Laboratory of Drug Research, Shanghai Institute of Materia Medica, Chinese Academy of Sciences, 555 Zu Chong Zhi Road, Zhangjiang Hi-Tech Park, Shanghai 201203, China*

* Corresponding authors: ywguo@simm.ac.cn (Y.-W.G.); xwli@simm.ac.cn (X.-W.L.); Tel.: +86-21-50806600-3317 (X.-W.L.)

Table of Contents

Table S1. EGFR inhibitory data of compounds **1-6** and the positive control staurosporine……...……...……...……...……...……...……...……...……...……...……...…………...…..4

Table S2. In silico docking parameters between compounds **2-6** and the ligand of 7XO site of 5X2A………………………………………………………………………………………....…..4

Figure S1. ^{1}H NMR spectrum of compound **1** (600 MHz, $CDCl_3$)……………………………………….5

Figure S2. ^{13}C NMR spectrum of compound **1** (125 MHz, $CDCl_3$) ……………………………………..5

Figure S3. DEPT135 spectrum of compound **1** (125 MHz, $CDCl_3$) ……………………………….……6

Figure S4. HSQC spectrum of compound **1** (600 MHz, $CDCl_3$) …………………………….…………..6

Figure S5. ^{1}H-^{1}H COSY spectrum of compound **1** (600 MHz, $CDCl_3$) ………………………………7

Figure S6. HMBC spectrum of compound **1** (600 MHz, $CDCl_3$) ………………………………..……7

Figure S7. NOESY spectrum of compound **1** (600 MHz, $CDCl_3$) ……………………………….………8

Figure S8. HR-ESI-MS spectrum of compound **1**…………………………………………………..………8

Figure S9. IR spectrum of compound **1**…………………………………………………………………..…9

Figure S10. ORD spectrum of compound **1**…………………………………...……………………………9

Figure S11. ^{1}H NMR spectrum of compound **2** (600 MHz, $CDCl_3$) ……………………..……………10

Figure S12. ^{13}C NMR spectrum of compound **2** (125 MHz, $CDCl_3$) …………………………….……10

Figure S13. DEPT135 spectrum of compound **2** (125 MHz, $CDCl_3$) ……………………………………11

Figure S14. HSQC spectrum of compound **2** (600 MHz, $CDCl_3$) ……………………………………………11

Figure S15. ^{1}H-^{1}H COSY spectrum of compound **2** (600 MHz, $CDCl_3$) ……………………….………12

Figure S16. HMBC spectrum of compound **2** (600 MHz, $CDCl_3$) ……………………………..…………12

Figure S17. NOESY spectrum of compound **2** (600 MHz, $CDCl_3$) ……………….………………………13

Figure S18. HR-ESI-MS spectrum of compound **2**……………………………………………………..…13

Figure S19. IR spectrum of compound **2**………………………………………………………….…………14

Figure S20. ORD spectrum of compound **2**……………………………….………………………………14

Figure S21. ^{1}H NMR spectrum of compound **3** (400 MHz, $CDCl_3$) ………………………………………15

Figure S22. ^{13}C NMR spectrum of compound **3** (125 MHz, $CDCl_3$) ………………..…………………15

Figure S23. ^{1}H NMR spectrum of compound **4** (400 MHz, $CDCl_3$) ………………………………………16

Figure S24. ^{13}C NMR spectrum of compound **4** (125 MHz, $CDCl_3$) ………………...…………………16

Figure S25. ^{1}H NMR spectrum of compound **5** (500 MHz, $CDCl_3$) ………………………………………17

Figure S26. ^{13}C NMR spectrum of compound **5** (125 MHz, $CDCl_3$) ……………………...……………17

Figure S27. ^{1}H NMR spectrum of compound **6** (500 MHz, $CDCl_3$) ………………………………………18

Figure S28. ^{13}C NMR spectrum of compound **6** (125 MHz, $CDCl_3$) ………………………….....……18

Figure S29. ^{13}H NMR spectrum of mixture (600 MHz, $CDCl_3$) …………………………….…………19

Figure S30. HR-ESI-MS spectrum of mixture…………………………………………………….………19
Figure S31. In silico binding mode of compounds **2-6** at EGFR kinase crystal structure 5X2A…………..20

Table S1. EGFR inhibitory data of compounds **1**–**6** and the positive control staurosporine.

Compound ID	Concentration (nM)	Mean	SD
**1**	20000	-9.9	4.5
**2**	20000	-12.5	4.2
**3**	20000	-3.8	5.3
**4**	20000	-3.4	5.8
**5**	20000	-7.5	3.1
**6**	20000	-4.9	3.7
**Staurosporine**	**IC_{50}** = 82.3 **nM**		

Table S2. In silico docking parameters between compounds **2-6** and the ligand of 7XO site of 5X2A

EGFR crystal structure	Compound ID	Number of hydrogen bonds	Binding Energy (kcal mol^{-1})	Van der Waals Energy (kcal mol^{-1})	-CDOCKER ENERGY (kcal mol^{-1})	-CDOCKER INTERACTION ENERGY (kcal mol^{-1})
5X2A	**2**	3	3.57	-19.62	-21.53	56.27
	**3**	1	1.83	-13.54	-28.67	47.16
	**4**	3	0.13	-14.00	-24.37	55.77
	**5**	1	8.52	-15.53	-25.47	54.58
	**6**	2	3.72	-14.21	-30.33	49.82

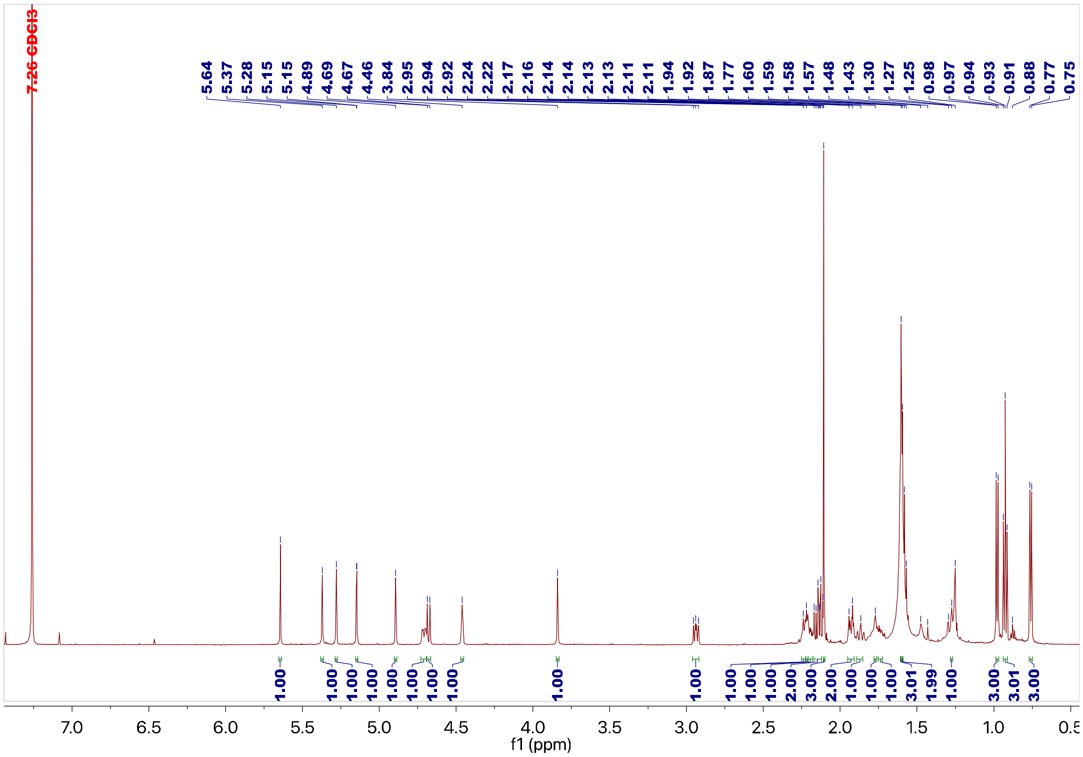

Figure S1. ^{1}H NMR spectrum of compound **1** in $CDCl_3$

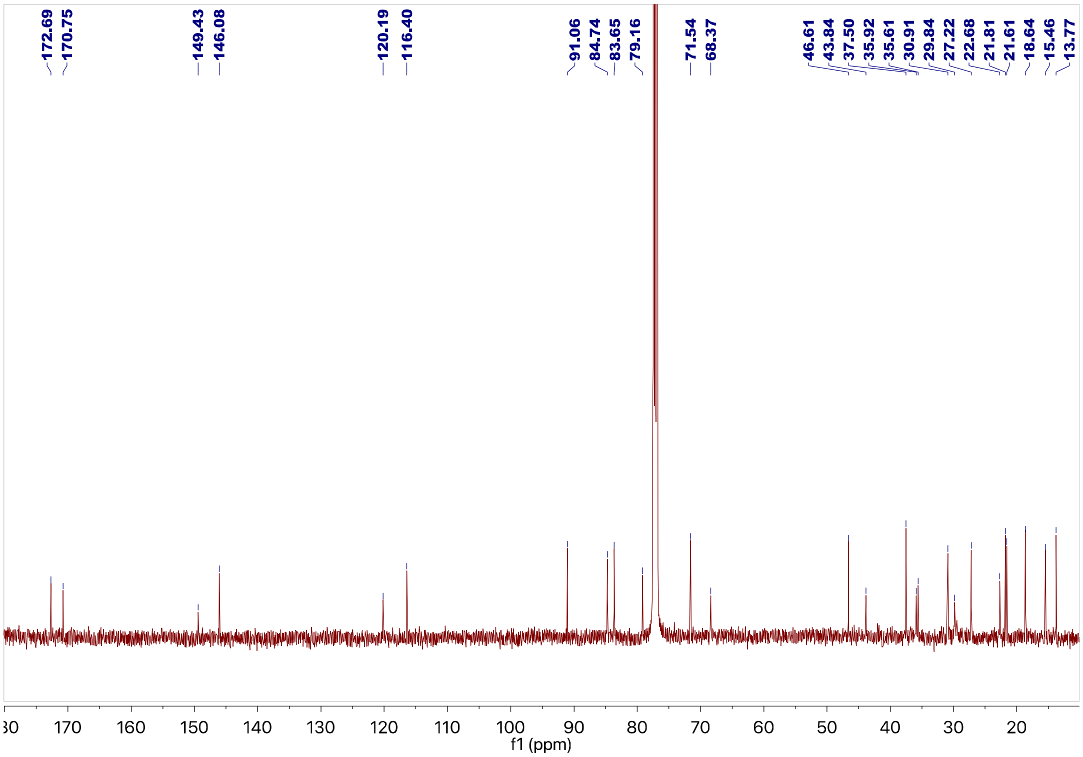

Figure S2. ^{13}C NMR spectrum of compound **1** in $CDCl_3$

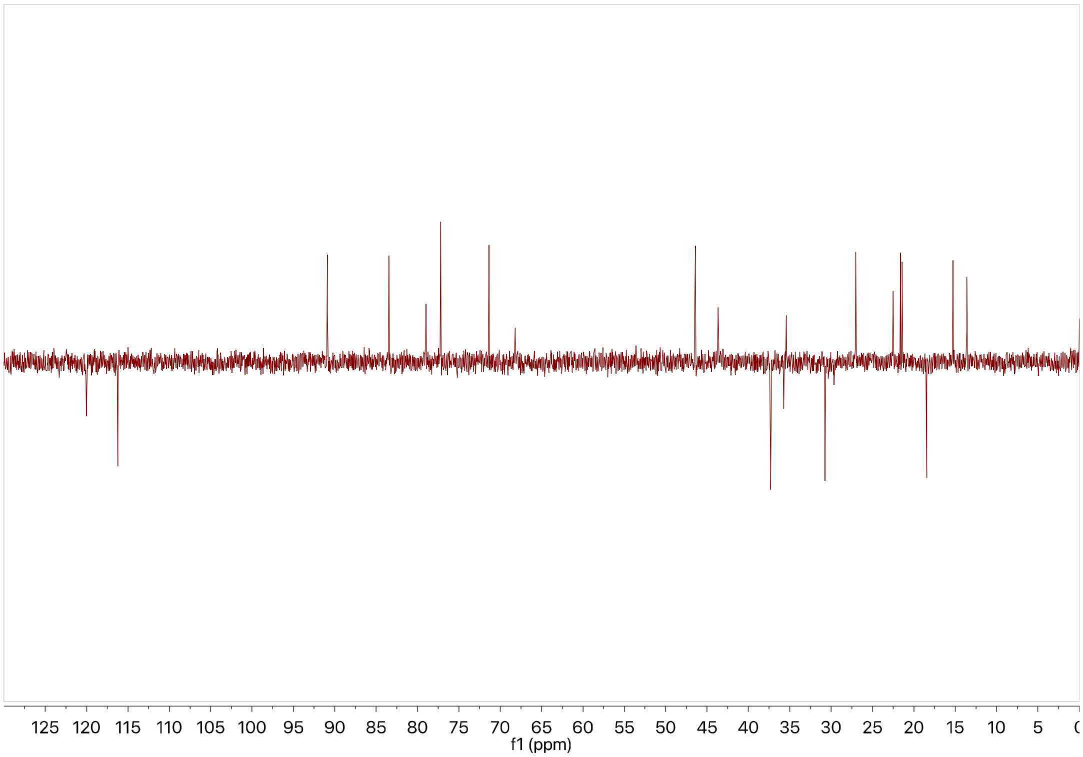

Figure S3. DEPT135 spectrum of compound **1** in $CDCl_3$

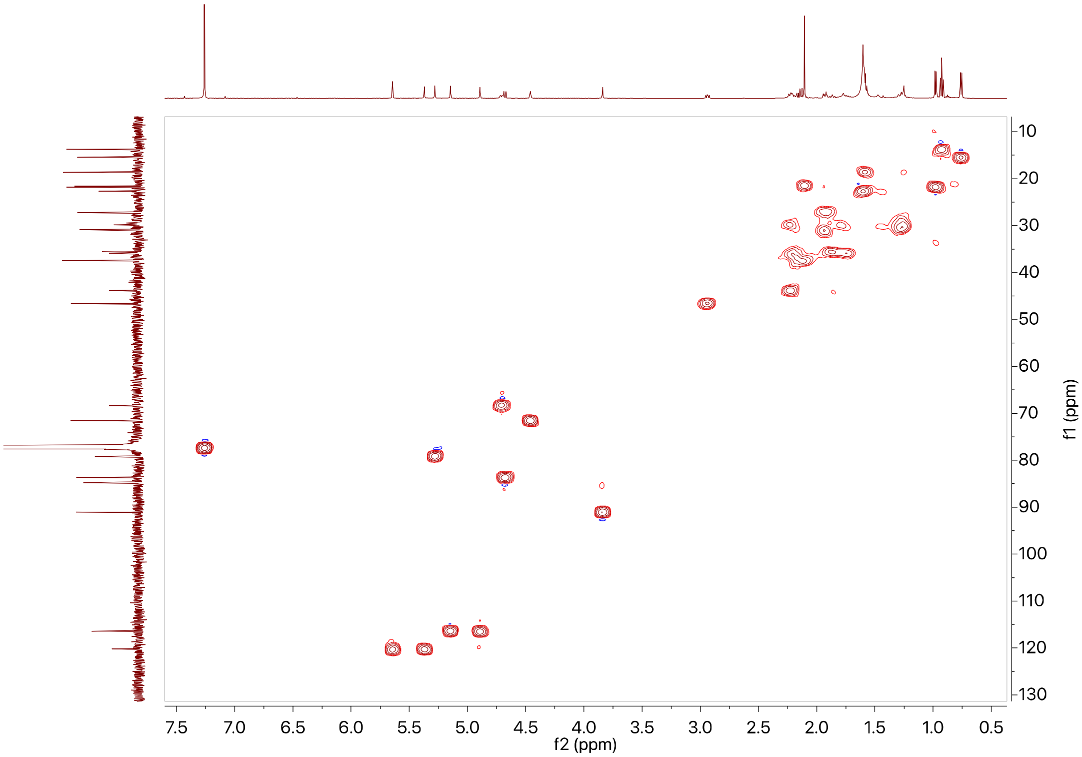

Figure S4. HSQC spectrum of compound **1** in $CDCl_3$

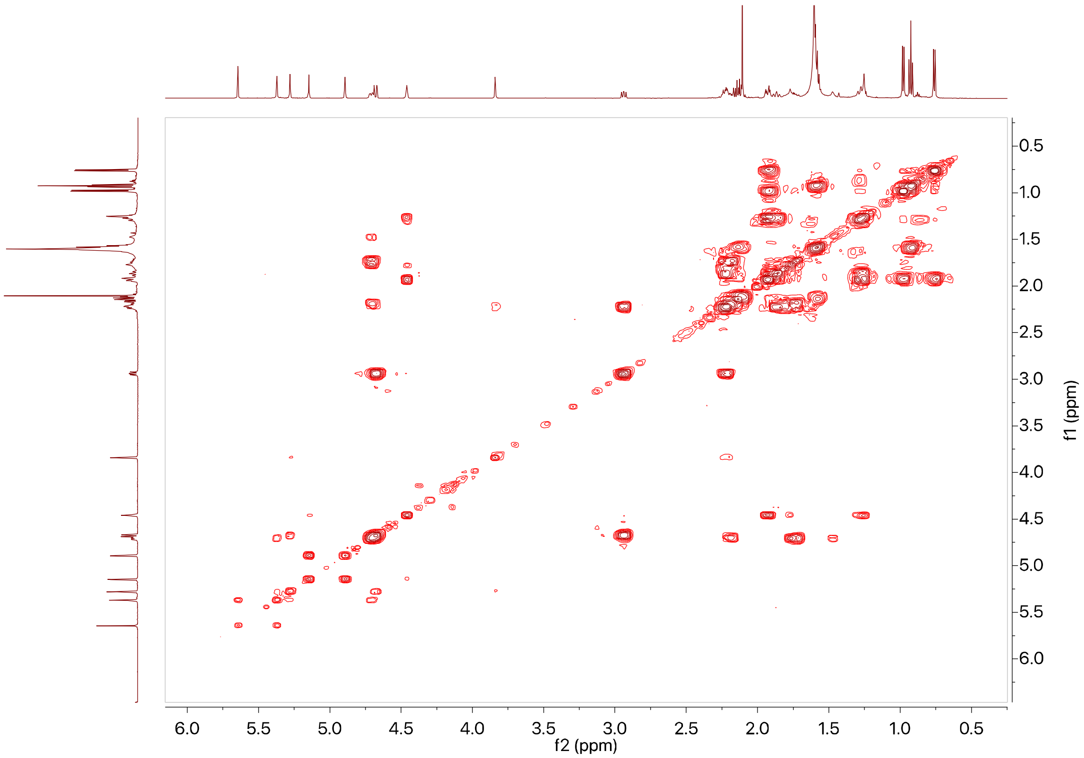

Figure S5. ^{1}H-^{1}H COSY spectrum of compound **1** in $CDCl_3$

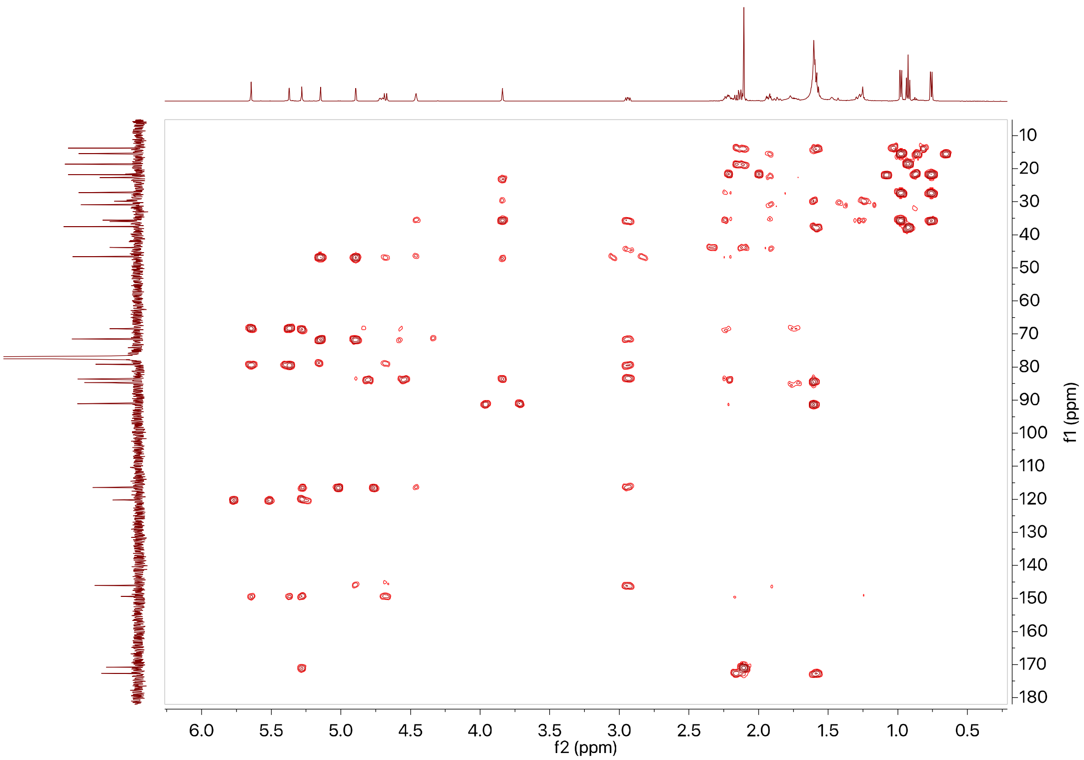

Figure S6. HMBC spectrum of compound **1** in $CDCl_3$

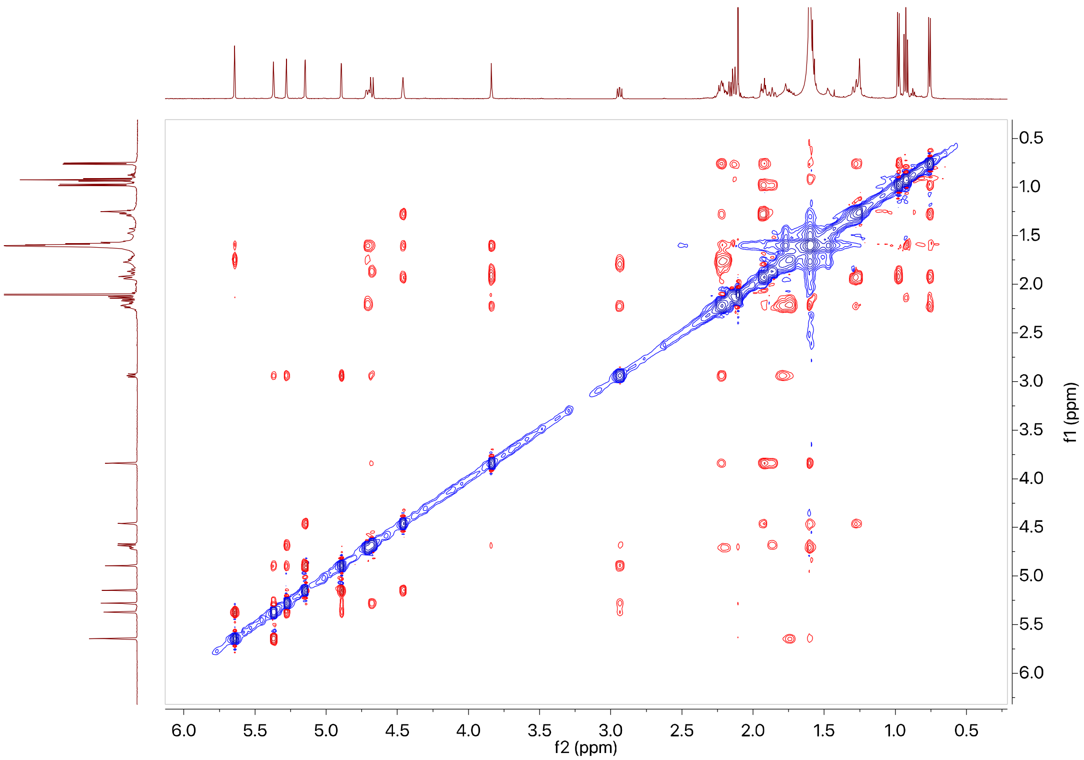

Figure S7. NOESY spectrum of compound **1** in $CDCl_3$

Qualitative Analysis Report

**Data Filename**	ESIH202103259.d	**Sample Name**	A8-A8-E3B
**Sample ID**		**Position**	P1-E3
**Instrument Name**	Agilent G6520 Q-TOF	**Acq Method**	20160322_MS_ESIH_POS_1min.m
**Acquired Time**	7/1/2021 19:56:48	**IRM Calibration Status**	Success
**DA Method**	small molecular data analysis method.m	**Comment**	ESIH by zhuzhenyun

User Spectra

Fragmentor Voltage	Collision Energy	Ionization Mode
135	0	ESI

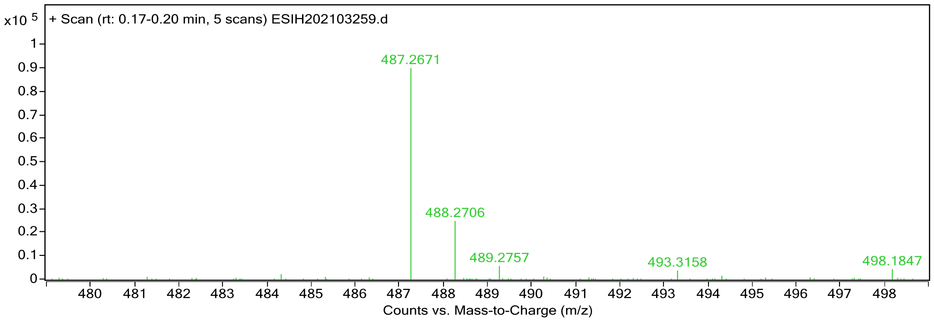

Formula Calculator Results

m/z	Calc m/z	Diff (mDa)	Diff (ppm)	Ion Formula	Ion
487.2671	487.2666	-0.47	-0.97	C26 H40 Na O7	(M+Na)+

--- End Of Report ---

Agilent Technologies　Page 1 of 1　Printed at: 17:22 on: 7/2/2021

Figure S8. HR-ESI-MS spectrum of compound **1**

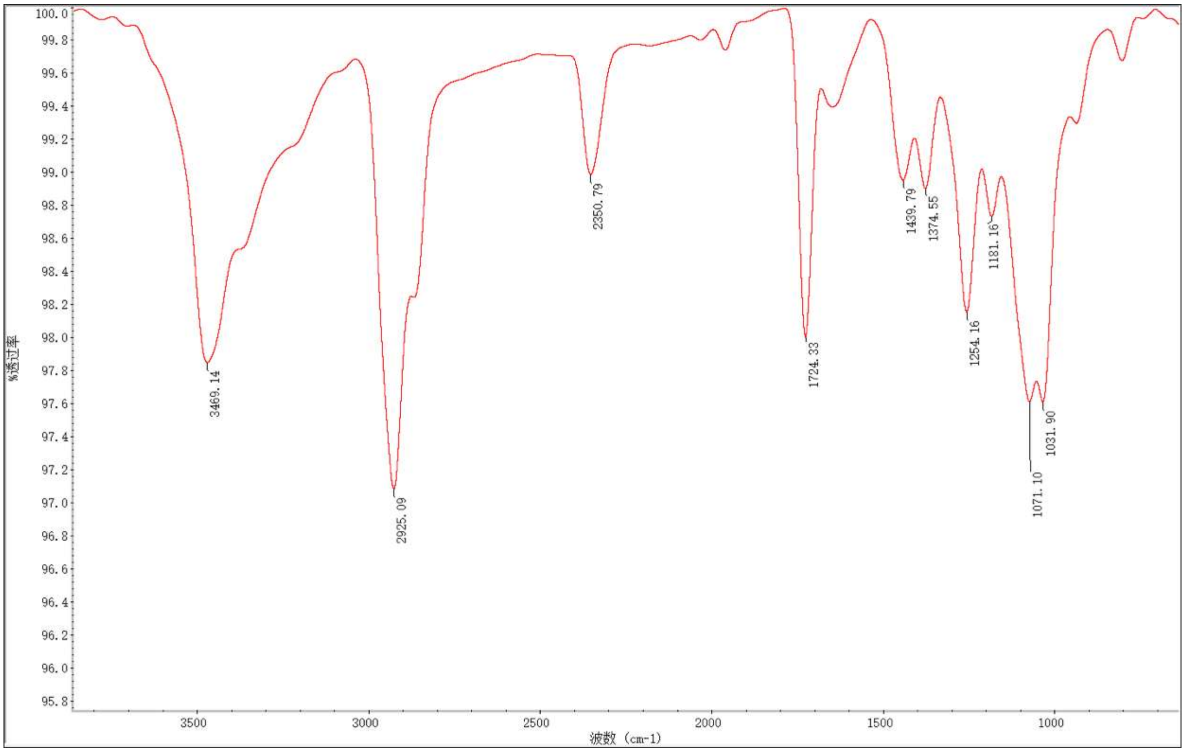

Figure S9. IR spectrum of compound **1**

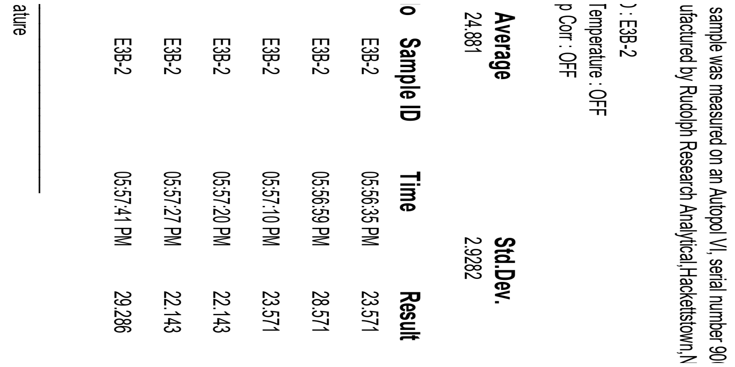
day, 06/28/2021
sample was measured on an Autopol VI, serial number 90
ufactured by Rudolph Research Analytical,Hackettstown,N
) : E3B-2
Temperature : OFF
p Corr : OFF

Average	Std.Dev.
24.881	2.9282

o	Sample ID	Time	Result
	E3B-2	05:56:35 PM	23.571
	E3B-2	05:56:59 PM	28.571
	E3B-2	05:57:10 PM	23.571
	E3B-2	05:57:20 PM	22.143
	E3B-2	05:57:27 PM	22.143
	E3B-2	05:57:41 PM	29.286

ature

Figure S10. ORD spectrum of compound **1**

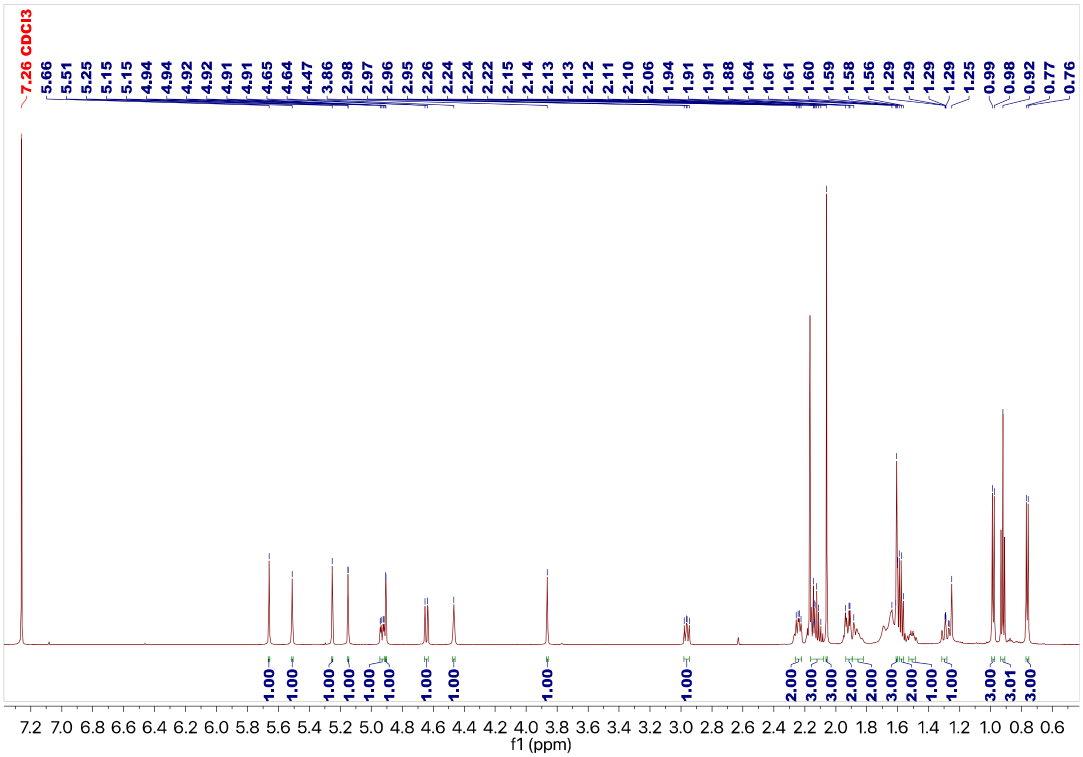

Figure S11. ^{1}H NMR spectrum of compound **2** in $CDCl_3$

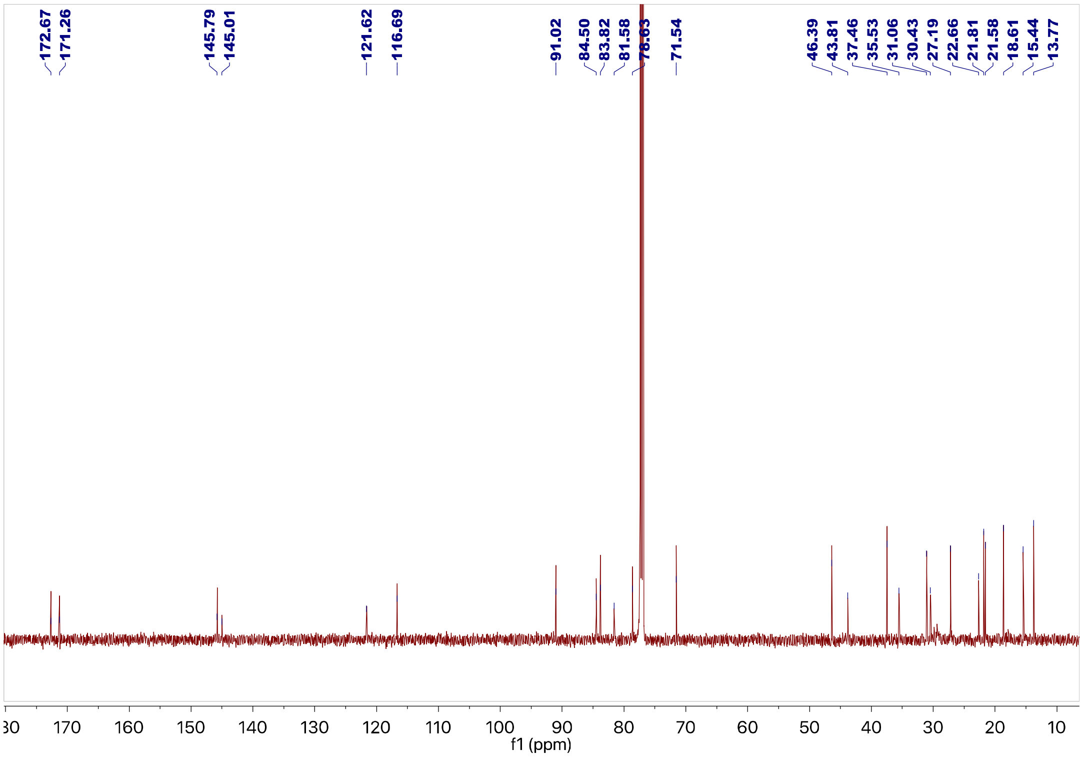

Figure S12. ^{13}C NMR spectrum of compound **2** in $CDCl_3$

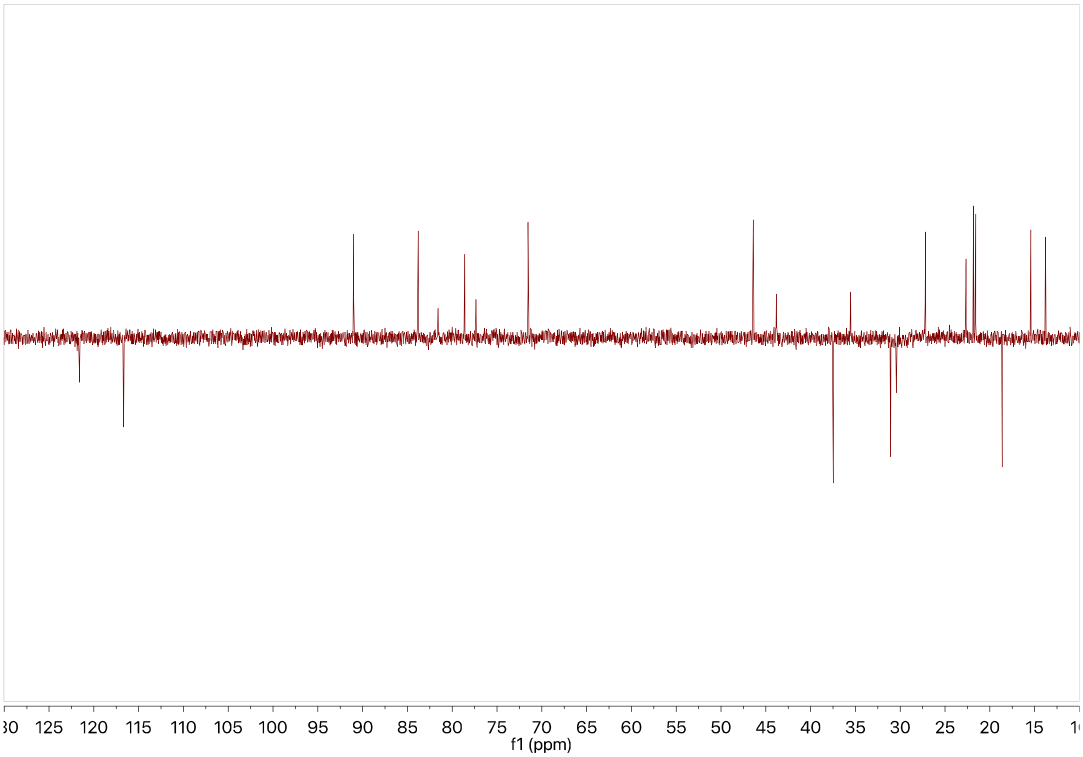

Figure S13. DEPT135 spectrum of compound **2** in $CDCl_3$

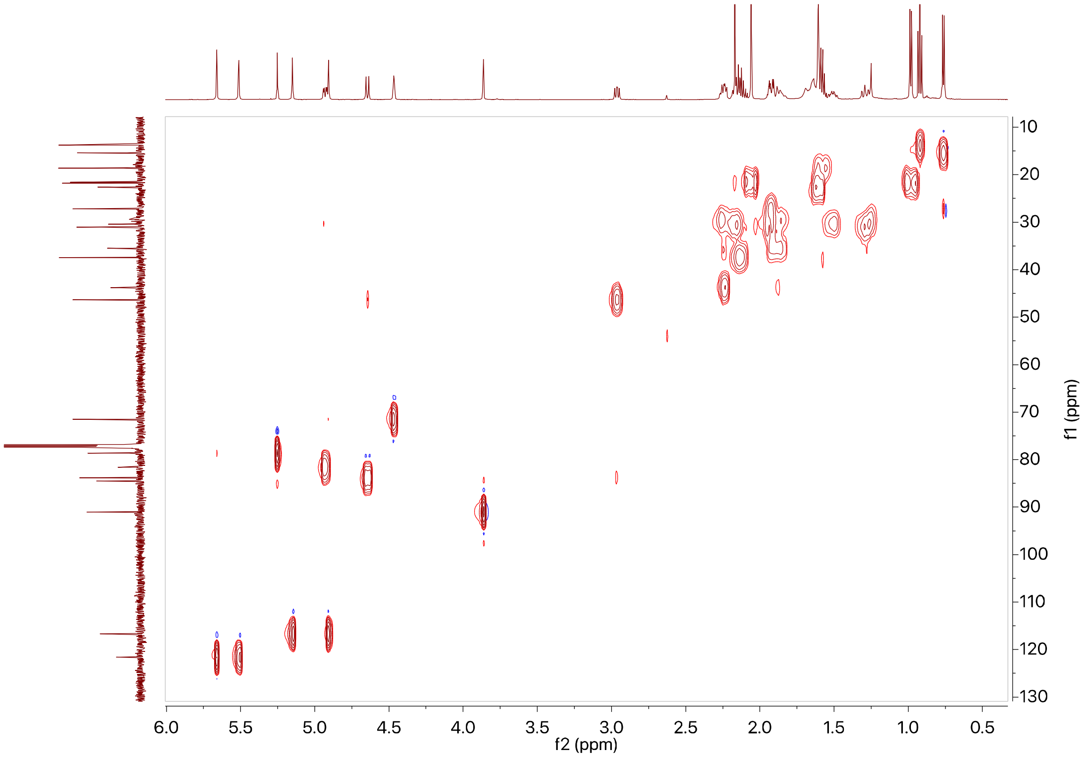

Figure S14. HSQC spectrum of compound **2** in $CDCl_3$

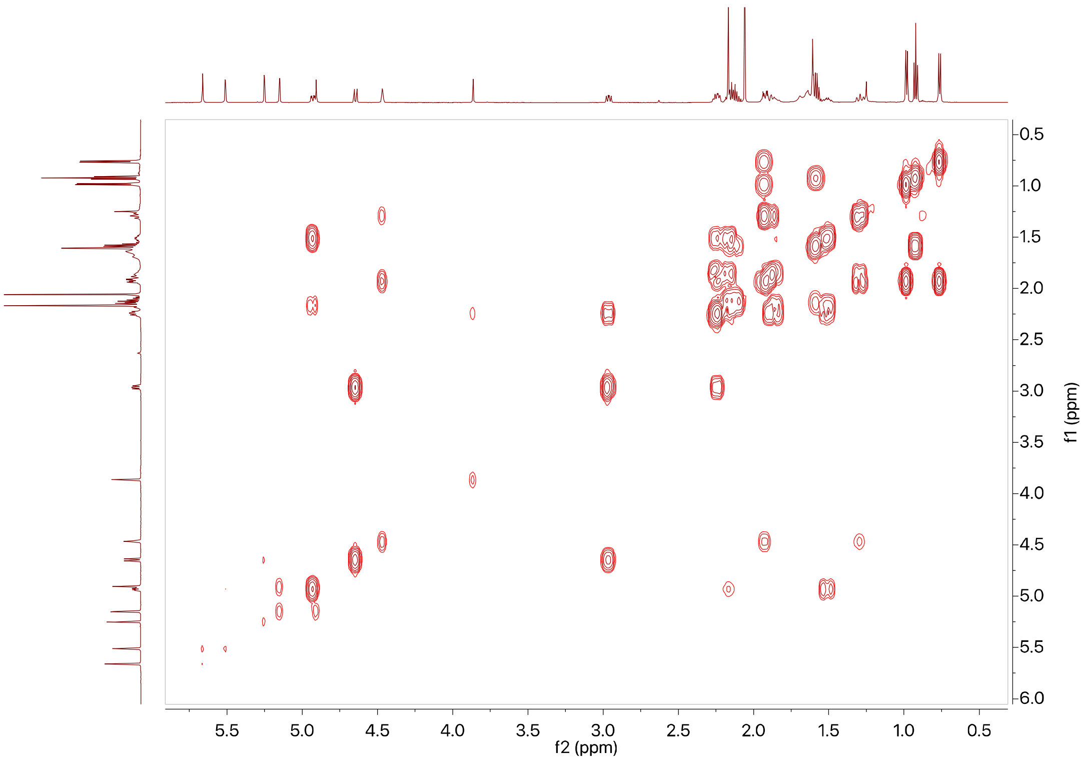

Figure S15. ^{1}H-^{1}H COSY spectrum of compound **2** in $CDCl_3$

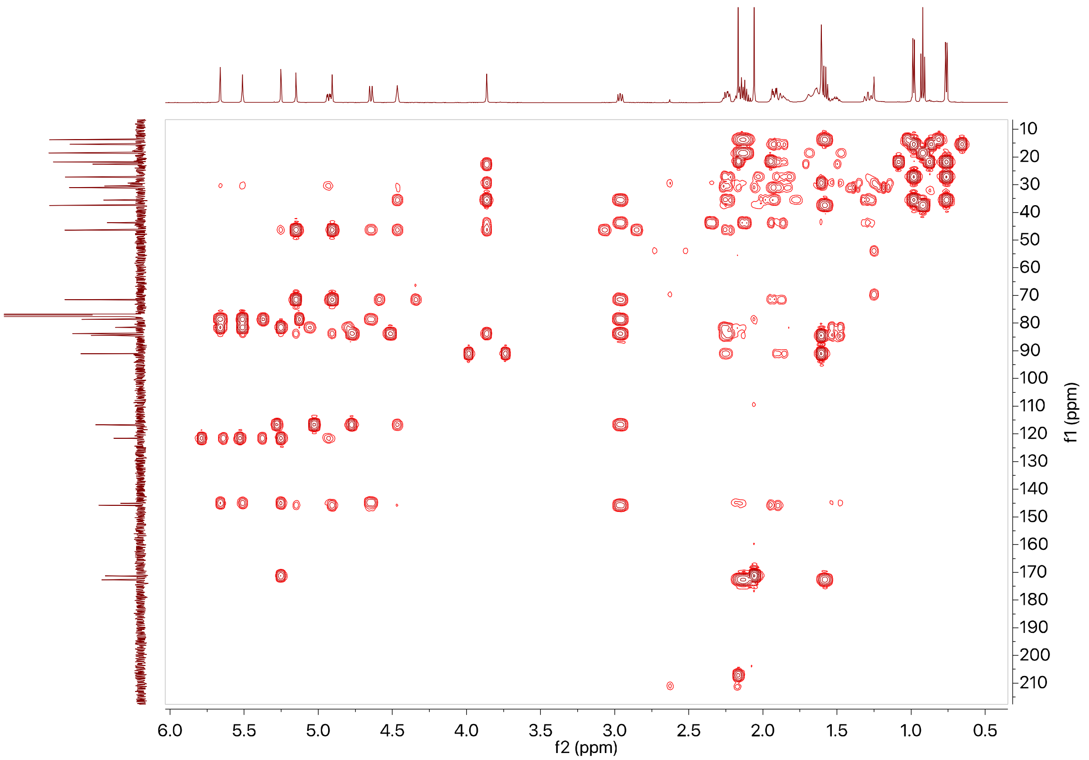

Figure S16. HMBC spectrum of compound **2** in $CDCl_3$

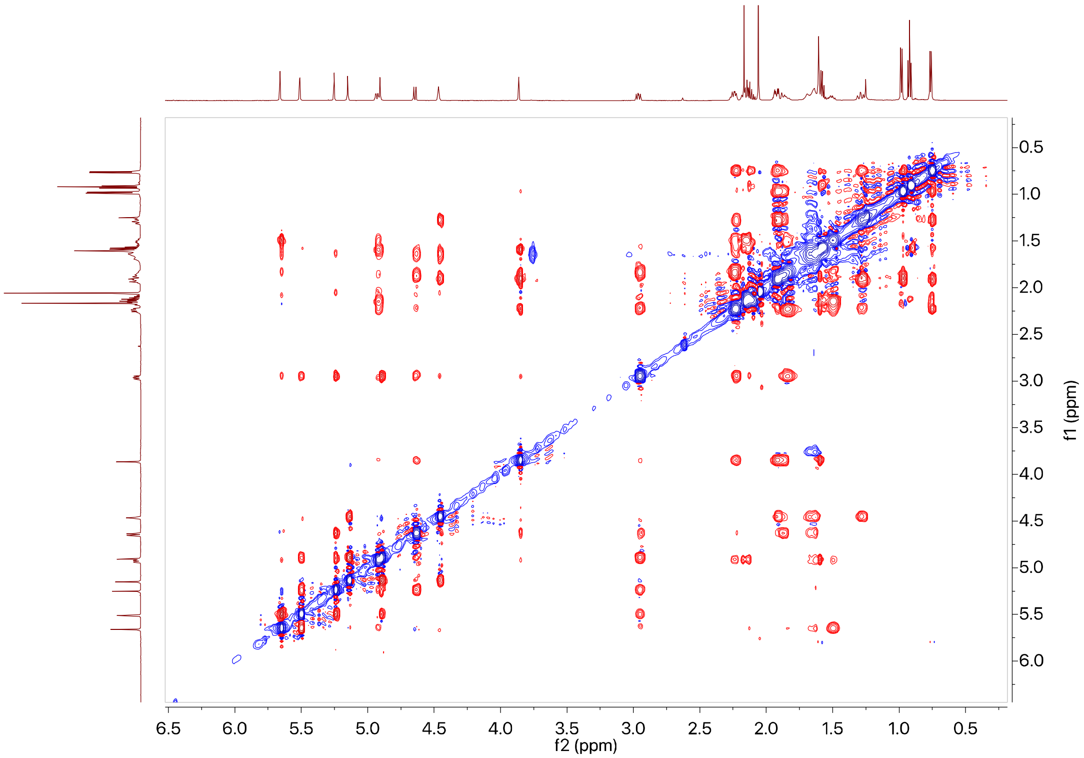

Figure S17. NOESY spectrum of compound **2** in $CDCl_3$

Qualitative Analysis Report

**Data Filename**	ESIH202102821.d	**Sample Name**	A8-A8-E4A
**Sample ID**		**Position**	P2-D2
**Instrument Name**	Agilent G6520 Q-TOF	**Acq Method**	20160322_MS_ESIH_POS_1min.m
**Acquired Time**	5/24/2021 17:23:13	**IRM Calibration Status**	Success
**DA Method**	small molecular data analysis method.m	**Comment**	ESIH by zhuzhenyun

User Spectra

Fragmentor Voltage	Collision Energy	Ionization Mode
135	0	ESI

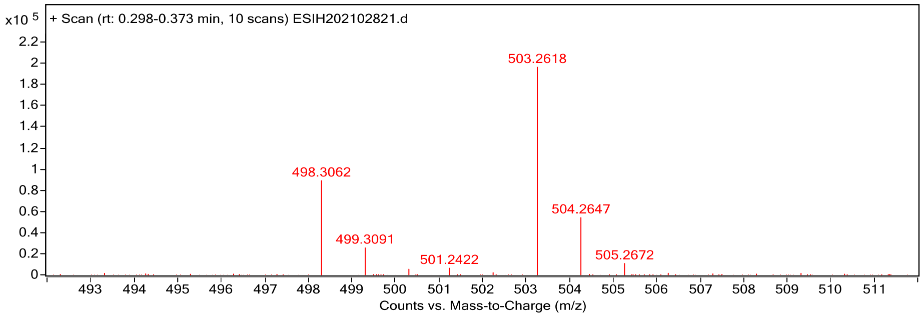

Formula Calculator Results

m/z	Calc m/z	Diff (mDa)	Diff (ppm)	Ion Formula	Ion
503.2618	503.2615	-0.31	-0.62	C26 H40 Na O8	(M+Na)+
498.3062	498.3061	-0.03	-0.05	C26 H44 N O8	(M+NH4)+

--- End Of Report ---

Agilent Technologies   Page 1 of 1   Printed at: 11:06 on: 5/25/2021

Figure S18. HR-ESI-MS spectrum of compound **2**

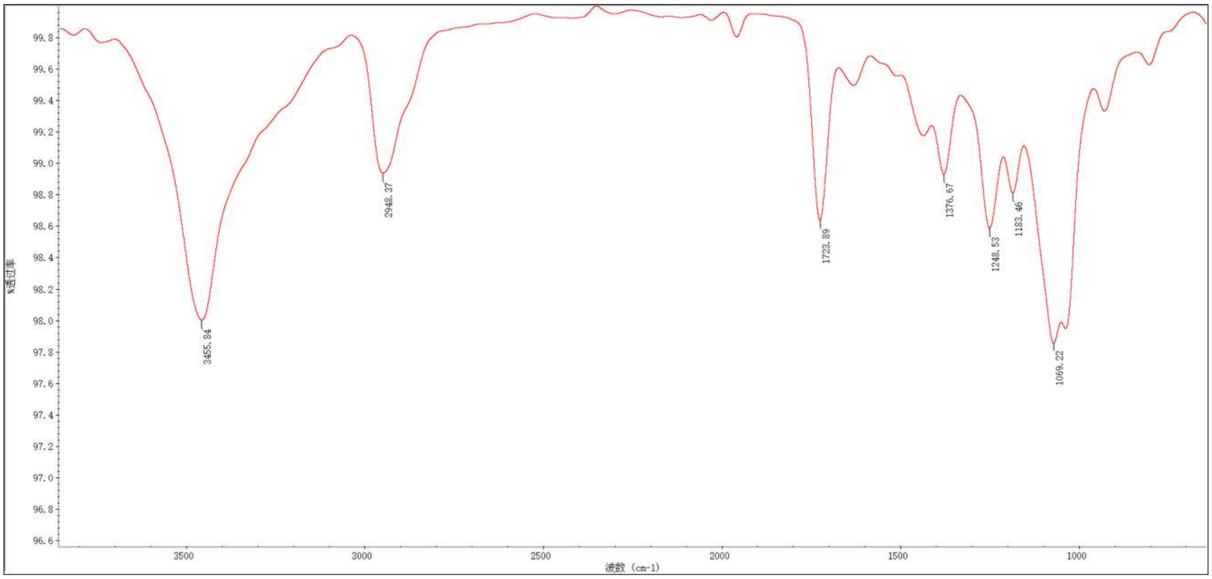

Figure S19. IR spectrum of compound **2**

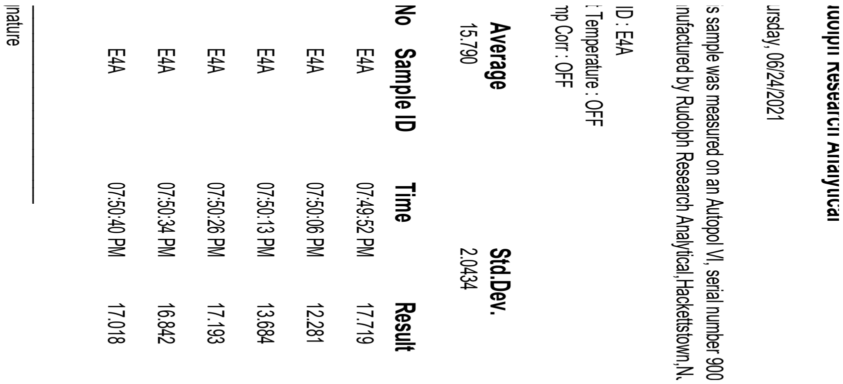

Figure S20. ORD spectrum of compound **2**

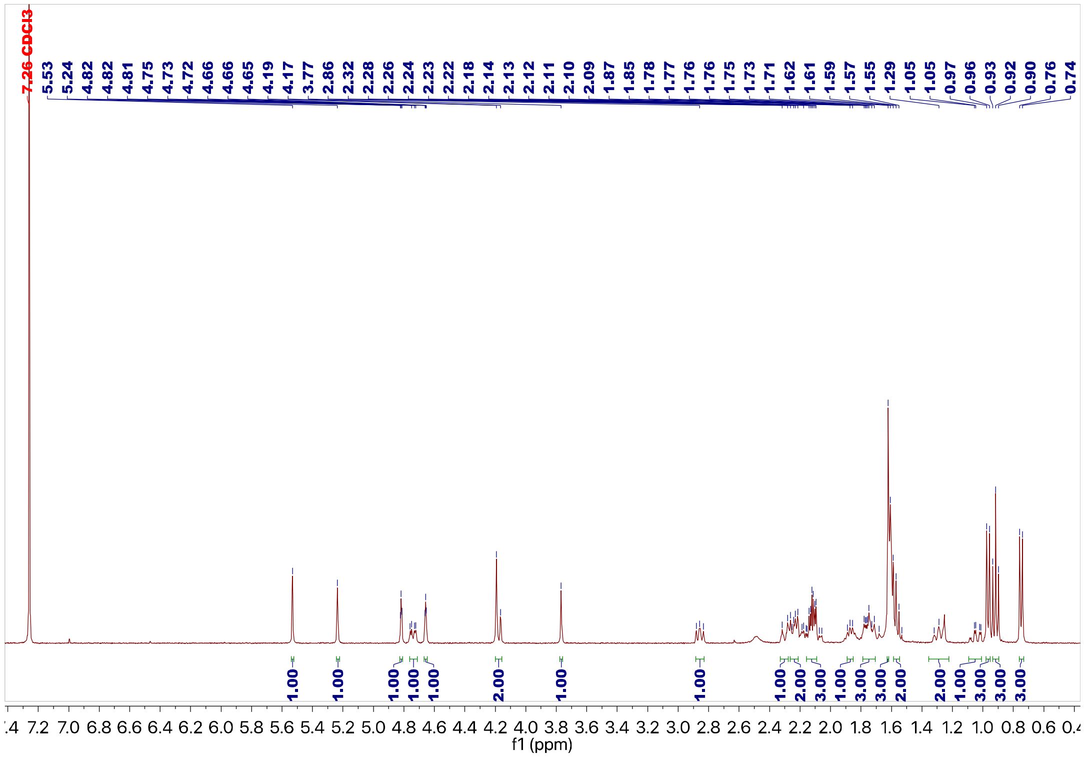

Figure S21. ^{1}H NMR spectrum of compound **3** in $CDCl_3$

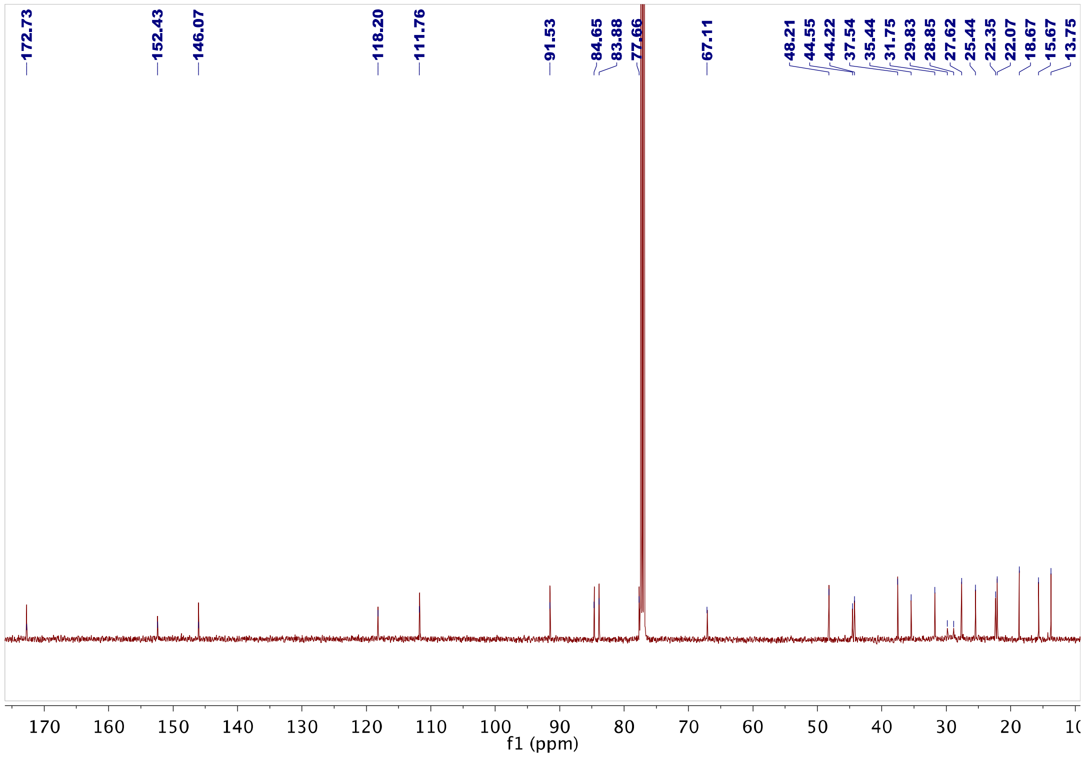

Figure S22. ^{13}C NMR spectrum of compound **3** in $CDCl_3$

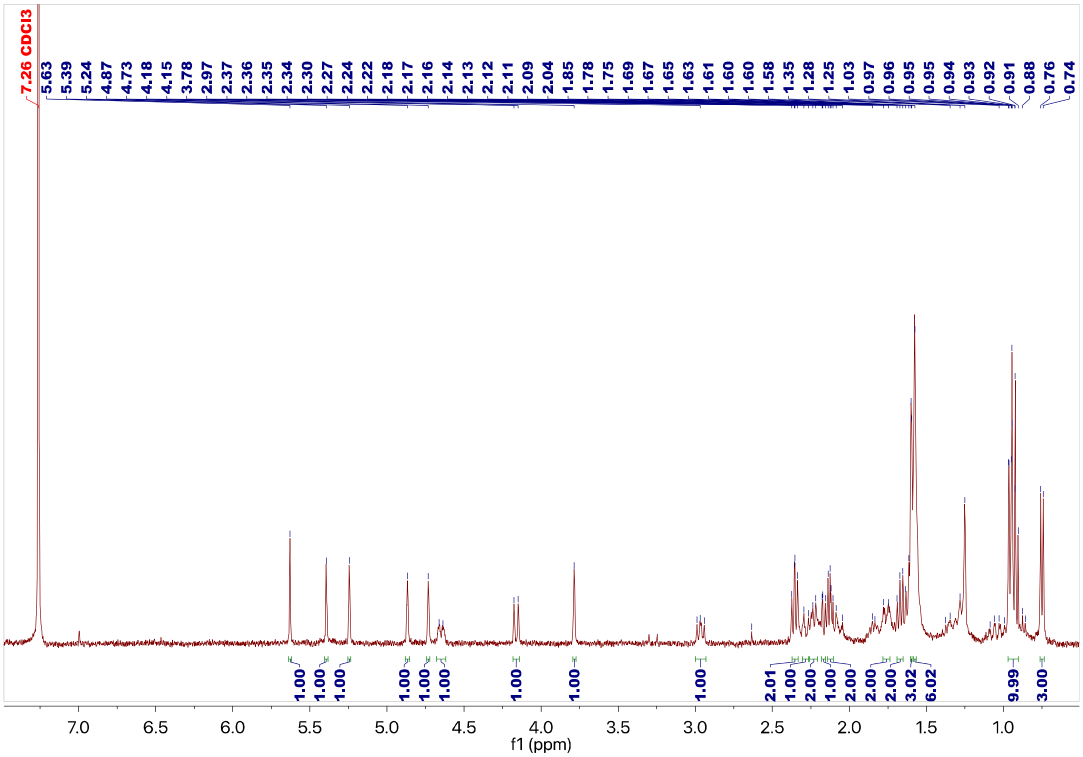

Figure S23. ^{1}H NMR spectrum of compound **4** in $CDCl_3$

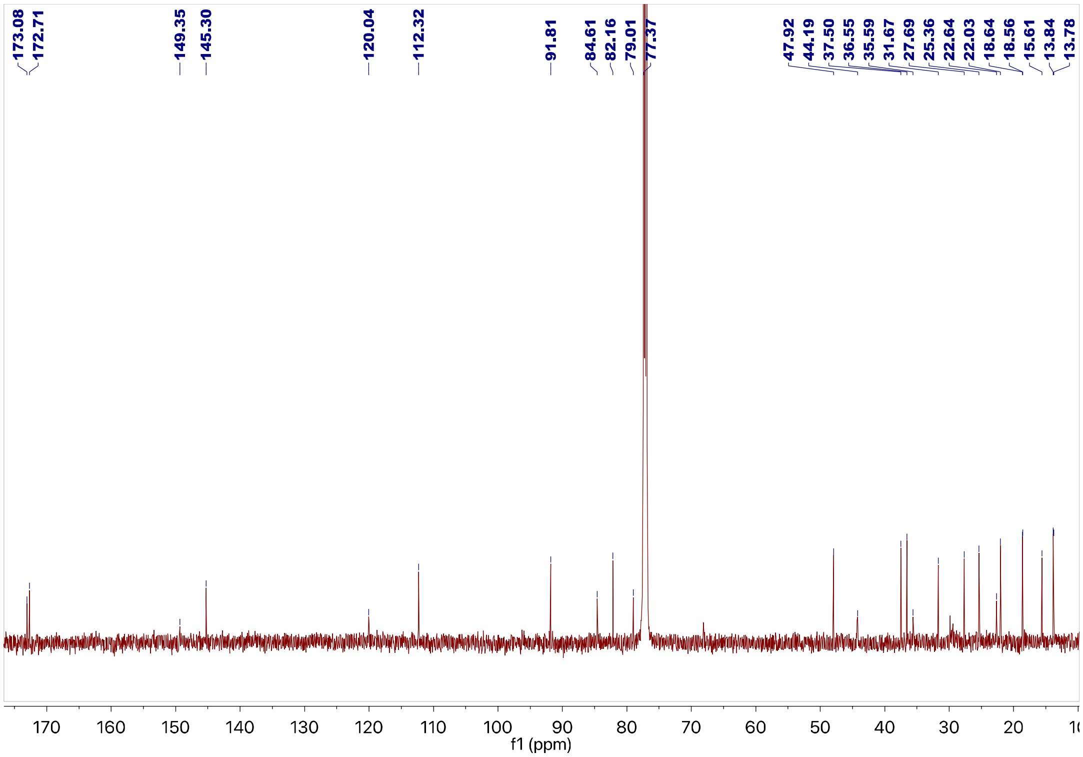

Figure S24. ^{13}C NMR spectrum of compound **4** in $CDCl_3$

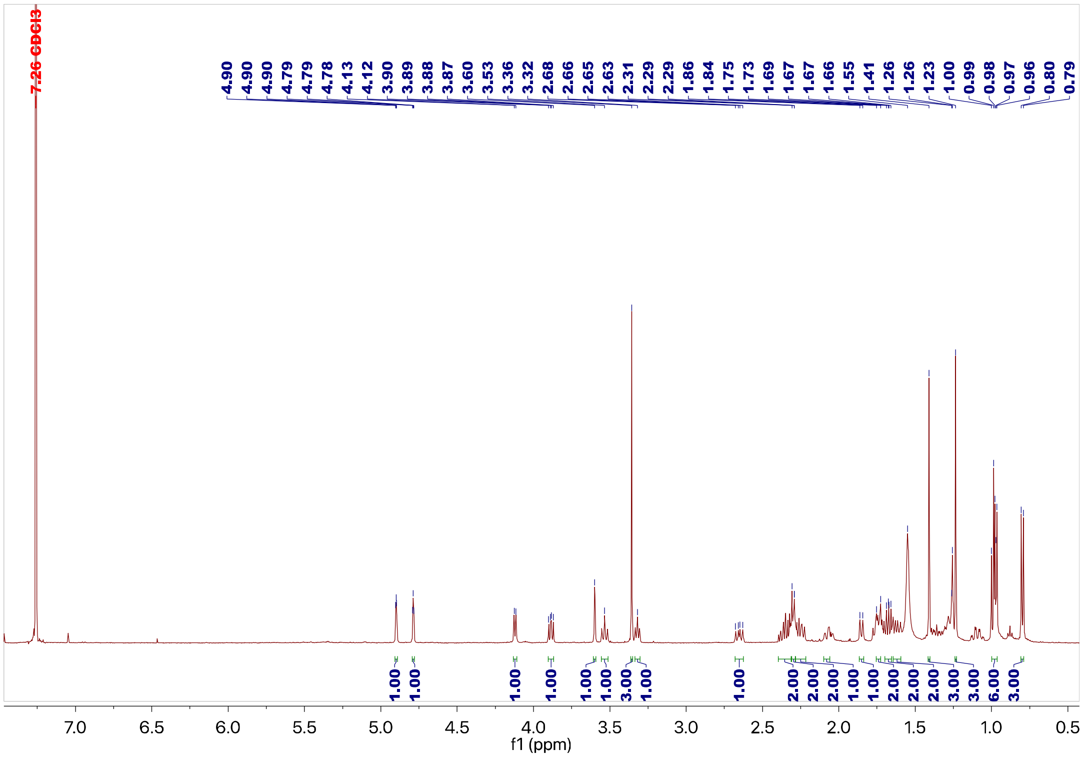

Figure S25. ^{1}H NMR spectrum of compound **5** in $CDCl_3$

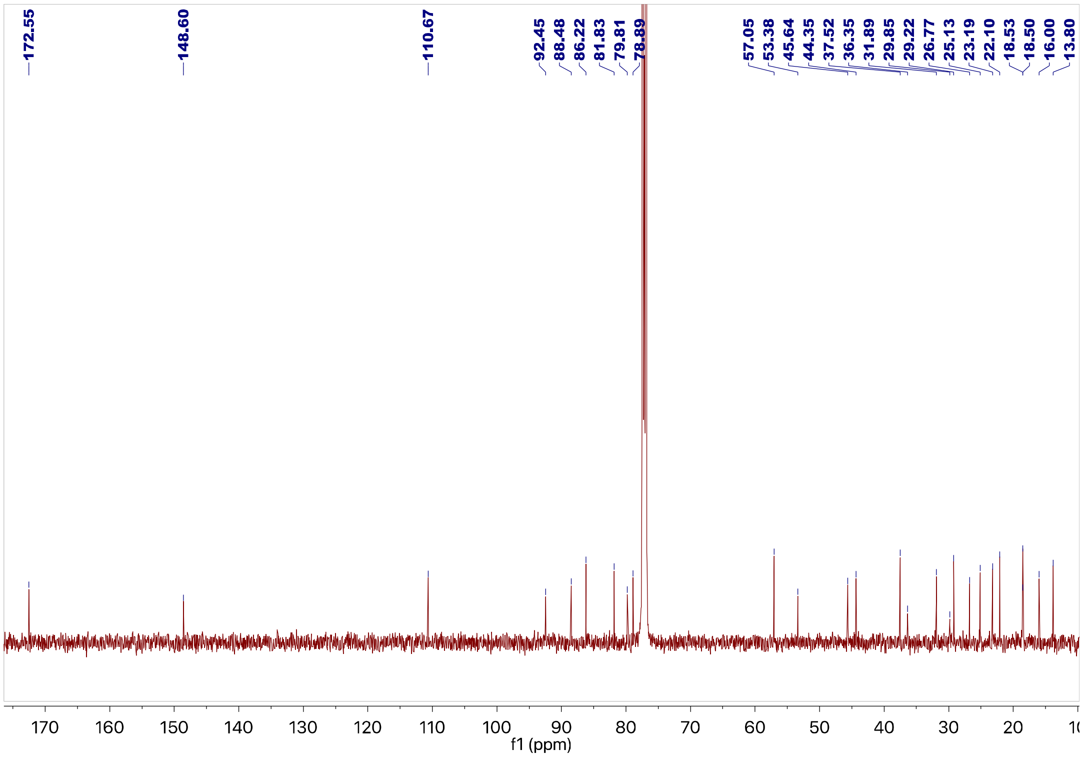

Figure S26. ^{13}C NMR spectrum of compound **5** in $CDCl_3$

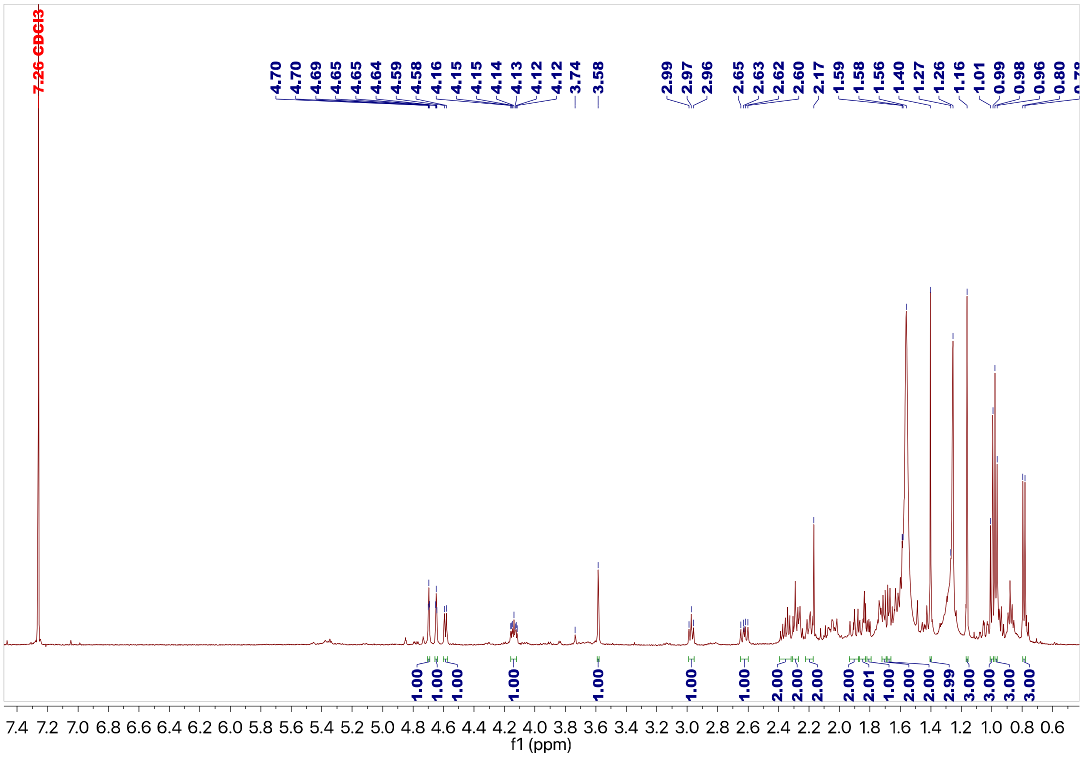

Figure S27. ^{1}H NMR spectrum of compound **6** in $CDCl_3$

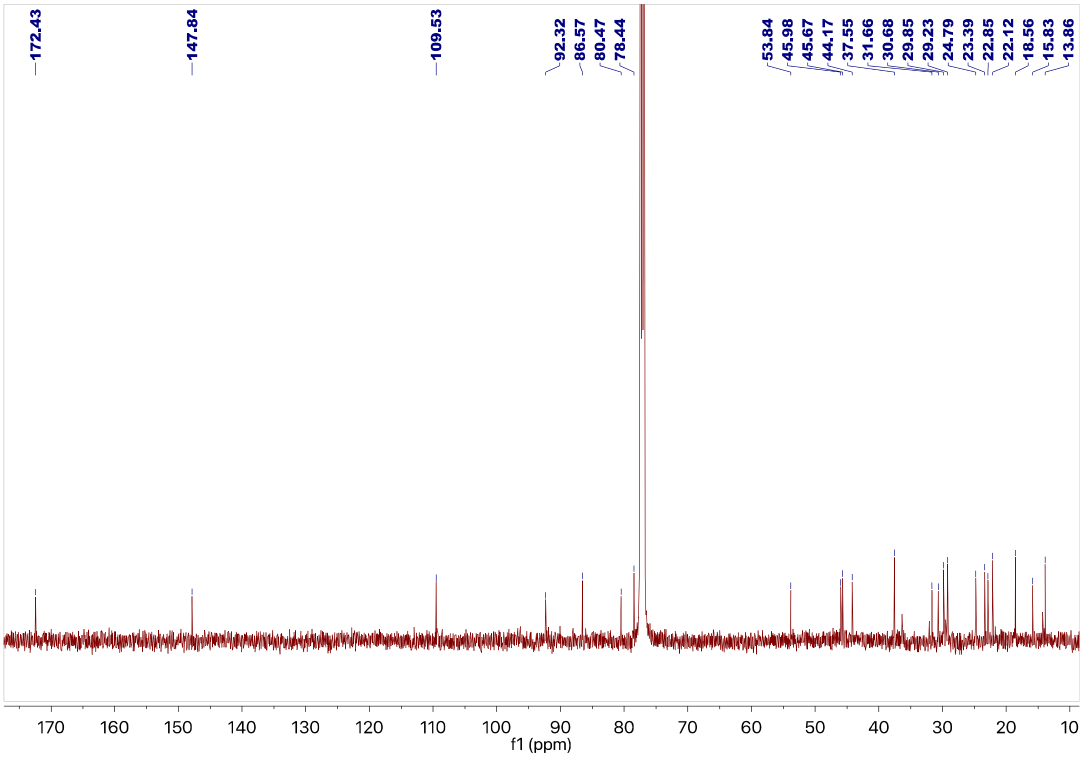

Figure S28. ^{13}C NMR spectrum of compound **6** in $CDCl_3$

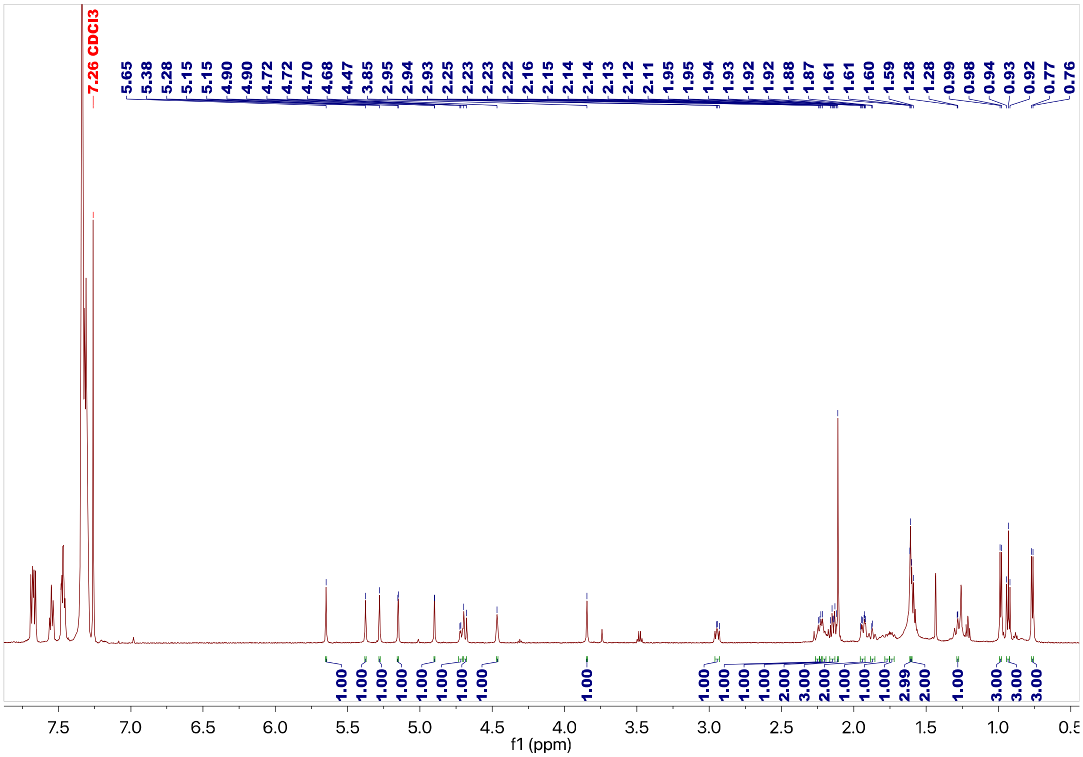

Figure S29. ^{1}H NMR spectrum of mixture in $CDCl_3$

Qualitative Analysis Report

**Data Filename**	ESIH202201014.d	**Sample Name**	A8-A8-E4A0302
**Sample ID**		**Position**	P1-B2
**Instrument Name**	Agilent G6520 Q-TOF	**Acq Method**	20160322_MS_ESIH_POS_1min.m
**Acquired Time**	3/2/2022 13:53:10	**IRM Calibration Status**	Success
**DA Method**	small molecular data analysis method.m	**Comment**	ESIH by fangsu

User Spectra

**Fragmentor Voltage**	**Collision Energy**	**Ionization Mode**
175	0	ESI

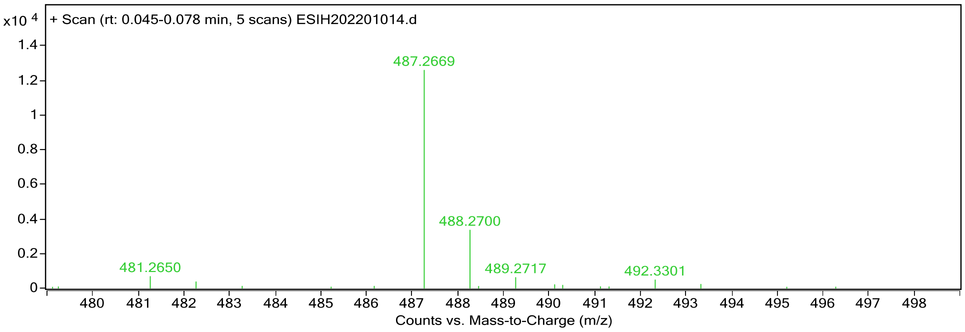

Formula Calculator Results

m/z	Calc m/z	Diff (mDa)	Diff (ppm)	Ion Formula	Ion
487.2669	487.2666	-0.27	-0.55	C26 H40 Na O7	(M+Na)+

--- End Of Report ---

Agilent Technologies   Page 1 of 1   Printed at: 14:43 on: 3/2/2022

Figure S30. HR-ESI-MS spectrum of mixture

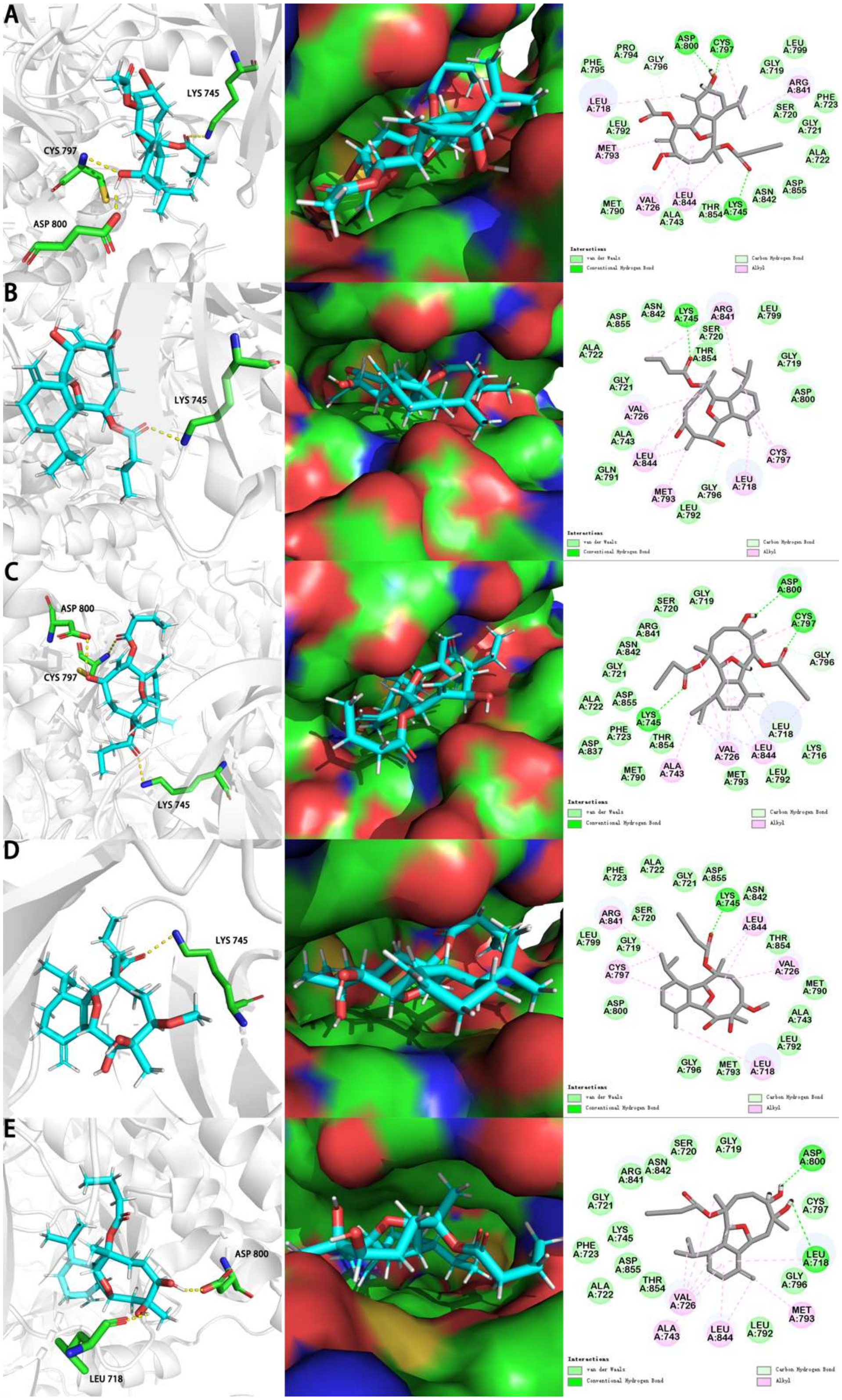

Figure S31. In silico binding mode of compounds **2–6** at EGFR kinase crystal structure 5X2A.
